# Penultimate deglacial warming across the Mediterranean Sea revealed by clumped isotopes in foraminifera

**DOI:** 10.1038/s41598-017-16528-6

**Published:** 2017-11-29

**Authors:** L. Rodríguez-Sanz, S. M. Bernasconi, G. Marino, D. Heslop, I. A. Müller, A. Fernandez, K. M. Grant, E. J. Rohling

**Affiliations:** 1 Research School of Earth Sciences, The Australian National University, Canberra, Australian Capital Territory, 2601 Australia; 2 Geological Institute, ETH Zurich, Sonneggstr. 5, 8092 Zurich, Switzerland; 3 Present Address: University of Vigo, Campus Universitario, 36310 Vigo, Spain; 4 Ocean and Earth Science, University of Southampton, National Oceanography Centre, Southampton, S014 3ZH UK

**Keywords:** Palaeoceanography, Palaeoclimate

## Abstract

The variability of seawater temperature through time is a critical measure of climate change, yet its reconstruction remains problematic in many regions. Mg/Ca and oxygen isotope (*δ*^18^O_C_) measurements in foraminiferal carbonate shells can be combined to reconstruct seawater temperature and *δ*^18^O (*δ*^18^O_SW_). The latter is a measure of changes in local hydrology (e.g., precipitation/evaporation, freshwater inputs) and global ice volume. But diagenetic processes may affect foraminiferal Mg/Ca. This restricts its potential in many places, including the Mediterranean Sea, a strategic region for deciphering global climate and sea-level changes. High alkalinity/salinity conditions especially bias Mg/Ca temperatures in the eastern Mediterranean (eMed). Here we advance the understanding of both western Mediterranean (wMed) and eMed hydrographic variability through the penultimate glacial termination (TII) and last interglacial, by applying the clumped isotope (*Δ*_47_) paleothermometer to planktic foraminifera with a novel data-processing approach. Results suggest that North Atlantic cooling during Heinrich stadial 11 (HS11) affected surface-water temperatures much more in the wMed (during winter/spring) than in the eMed (during summer). The method’s paired *Δ*_47_ and *δ*^18^O_C_ data also portray *δ*^18^O_SW_. These records reveal a clear HS11 freshwater signal, which attenuated toward the eMed, and also that last interglacial surface warming in the eMed was strongly amplified by water-column stratification during the deposition of the organic-rich (sapropel) interval known as S5.

## Introduction

Sedimentary sequences from the Mediterranean Sea are excellent paleoclimate archives to resolve outstanding questions on Earth’s climate system, due to a unique understanding of terrestrial, oceanic, and atmospheric interactions in the basin^1–4^. So far, this has allowed (i) long term records of sea-level changes to be generated by exploiting the sensitivity of the seawater oxygen composition (*δ*^18^O_SW_, reflected in the oxygen stable isotope records of planktic foraminifera, *δ*^18^O_C_) to global sea-level variations^5^, and (ii) marine sediment records to be placed on absolute chronologies by using nearby speleotherm records^2,6^.

Although these approaches have substantially contributed to understanding of past climate changes^2,5–7^, the full potential of Mediterranean sequences as paleoclimate archives remains to be exploited because of problems with sea surface temperature reconstructions in the Mediterranean Sea using well-stablished proxies. First, the alkenone-based proxy (${{\rm{U}}}_{37}^{{\rm{K}}^{\prime} }$, ref.^8^) may be excellent for reconstructing temperatures in the wMed, but is limited in the eMed by poor preservation of organic matter^9^. Also, alkenone-based reconstructions do not necessarily reflect the conditions (depth, season) in which the key planktic foraminiferal species calcified, which hinders the precision of corrections for the temperature component of *δ*^18^O_C_, to resolve *δ*^18^O_SW_. Second, the foraminiferal Mg/Ca method, which allows simultaneous reconstruction of temperature and *δ*^18^O_SW_ using the same signal carrier^10^, is compromised under high alkalinity/salinity conditions, such as those in the eMed^11^ where conventional foraminiferal analyses are problematic^12,13^.

Here we use new developments concerning the clumped isotope paloethermometer^14–16^ (*Δ*_47_) to reconstruct temperatures and deconvolve *δ*^18^O_SW_ in the Mediterranean Sea, through paired *Δ*_47_-*δ*^18^O_C_ measurements in foraminiferal calcite^17^. The *Δ*_47_ relates the abundance of ^13^C-^18^O bonds in the calcite lattice to the temperature at which the calcite precipitates^16^ and increases as precipitation temperature decreases. This proxy is attractive for palaeoclimatic applications because: (*i*) state-of-the-art analytical instrumentation indicates that the *Δ*_47_-values of inorganic and biogenic calcites precipitated at the same temperature are consistent with one-another^18–20^, which suggests negligible vital effects^15,18^; and (*ii*) it does not require information on the chemistry of the seawater in which the foraminifera calcified^15^.

The temperature sensitivity of the *Δ*_47_ proxy is low^19^ (~0.003‰/°C). It therefore requires high measurement precision (~0.01‰, 1SE), which is commonly achieved by increasing counting times and/or the number of replicates analysed per sample^21,22^. While this is feasible for applications that use large carbonate samples (~5 mg per replicate), it is not realistic for foraminifer-based reconstructions because many hundreds of specimens would be required for a single measurement. However, recent development of a clumped isotope methodology (*Δ*_47_-small method^23–26^) has reduced the sample size for a precise *Δ*_47_ measurement, and thus made the proxy amenable for temperature reconstructions based on foraminifera^17,27^. The *Δ*_47_-small method uses a series of replicate carbonate analyses (~150 μg each *Δ*_47_-replicate) to perform multiple paired *Δ*_47_-*δ*^18^O_C_ measurements, and combinations of these then yield average temperature and *δ*^18^O_SW_ values^17^. The method considers that the *Δ*_47_ precision improves by increasing the number of paired *Δ*_47_-*δ*^18^O_C_ measurements from the same and/or neighbour samples, and the potential of this approach for palaeoclimate reconstructions has been demonstrated previously^17,28^. However, there have been only few foraminiferal *Δ*_47_ records from this approach, with only few datapoints^17,28^. For broad utility in palaeoclimate research, more continuous records are needed to facilitate reconstructions of phase relationships between climatic forcing(s) and response(s).

Here we expand on the analytical improvements of the *Δ*_47_-small method^24,25^ by means of a new data-analysis approach that allows expressing *Δ*_47_-small data in records of much improved continuity (hereafter: non-traditional data-analysis). We use the non-traditional data-analysis approach to provide paired temperature and *δ*^18^O_SW_ reconstructions from the wMed and the eMed. We target the penultimate glacial termination (TII) and the last interglacial period because this interval comprises two important climatic events: Heinrich Stadial 11 (HS11; 135–130 ka refs^2,29^) and Sapropel S5 (128.3–121.5 ka, ref.^30^). Using existing techniques, it has not been possible to assess the spatial impacts of these events on the Mediterranean hydrography. Moreover, reconstructions based on ${{\rm{U}}}_{37}^{{\rm{K}}^{\prime} }$ across this interval in the wMed show a consistent temperature evolution with a cooling step of 4 ± 2 °C (1σ) during HS11 (refs^2,29^), followed by warming of 11 ± 2 °C starting at ~130 ka (ref.^29^). These are ideal targets for testing our new foraminiferal *Δ*_47_-approach.

## Results

### Paired *Δ*_47_-*δ*^18^O_C_ measurements in the western Mediterranean Sea

**Figure 1:**
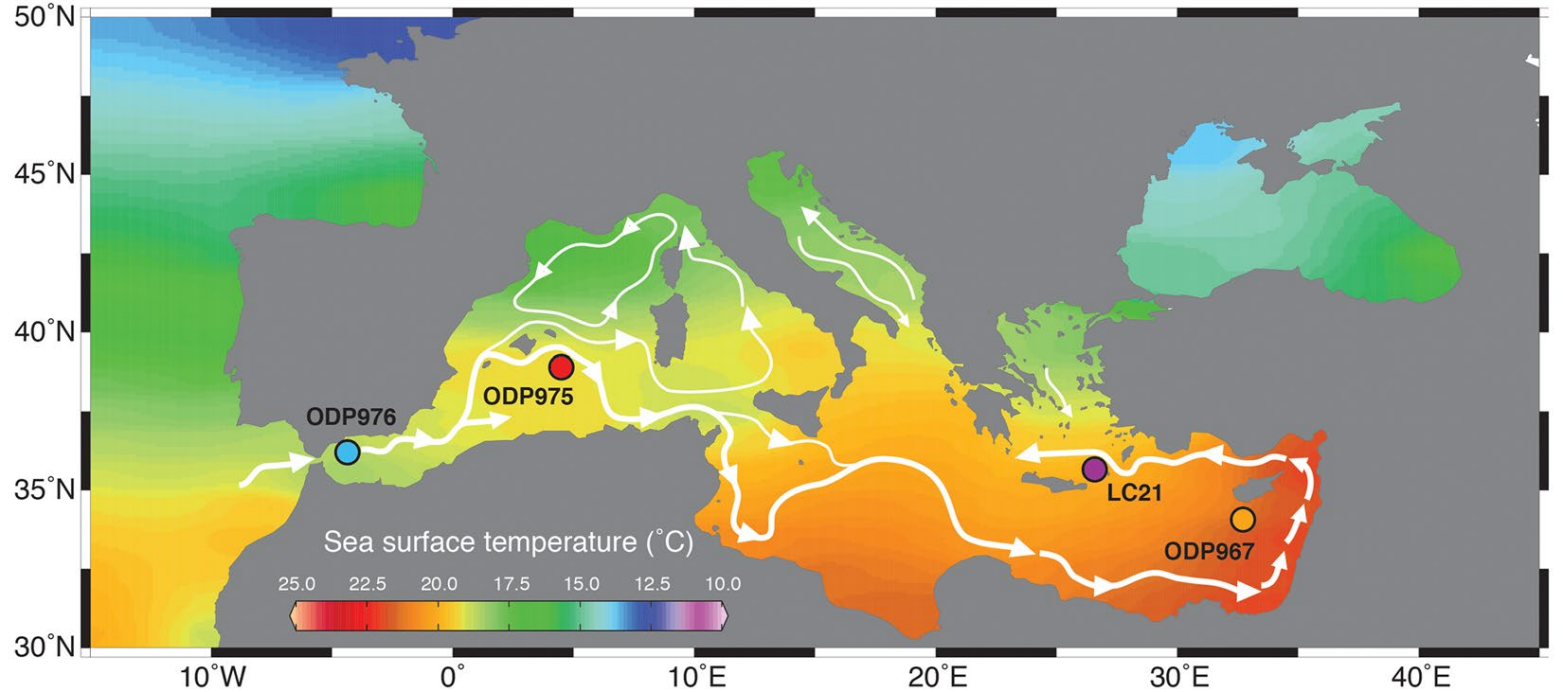
Map and sediment core location. Mean annual sea surface temperature contour map created using http://odv.awi.de/ (ref.^57^) of the Mediterranean Sea with core locations (dots) and a schematic view of surface circulation.

**Figure 2:**
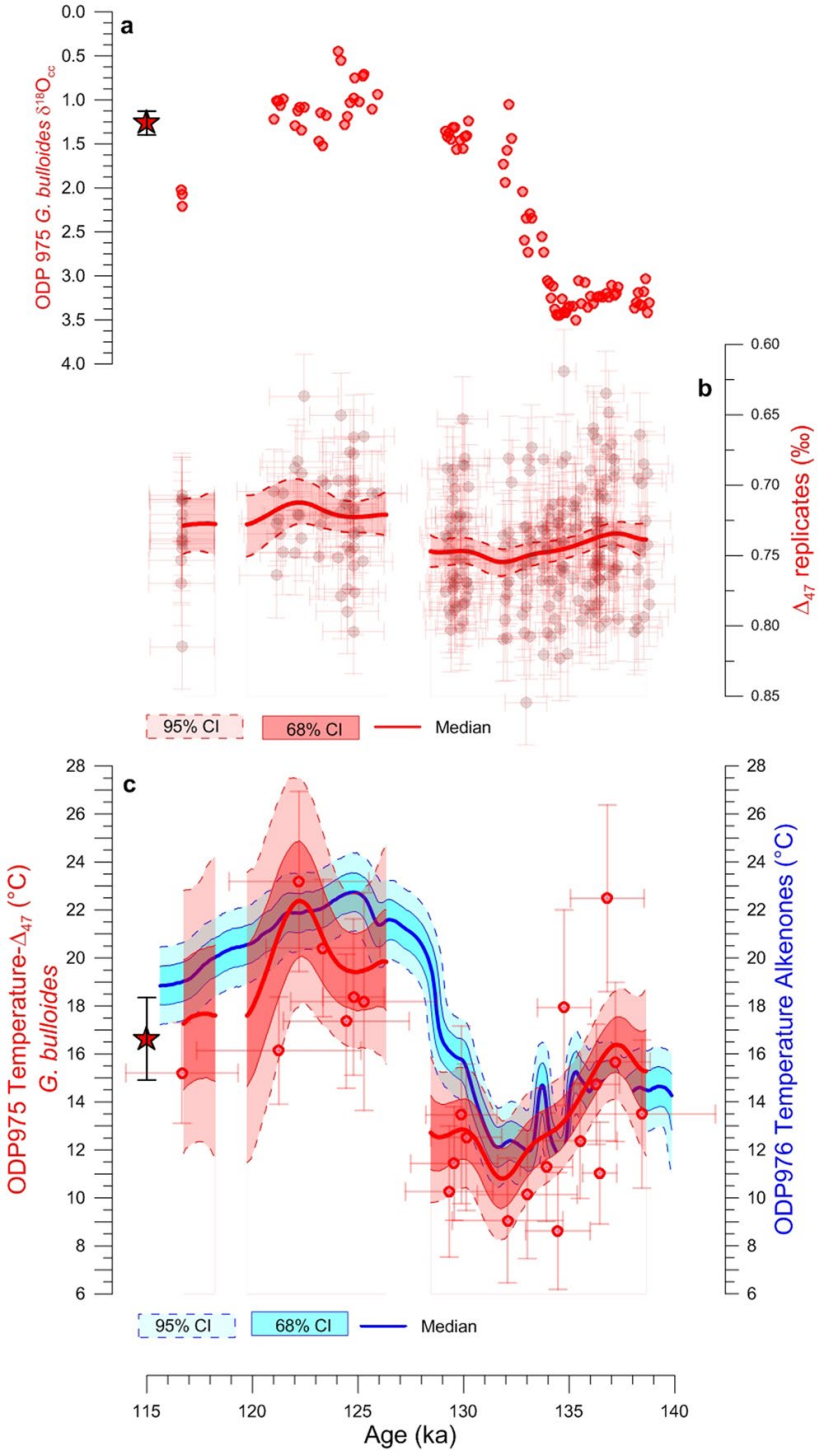
Western Mediterranean temperature records across TII. ODP975 *G*. *bulloides* (**a**) *δ*^18^O_C_ and (**b**) *Δ*_47_-replicates. (**c**) ODP975 *G*. *bulloides Δ*_47_-temperature record (red, Methods) using conventional *Δ*_47_-binning (dots) and non-traditional data-analysis approach (the median and the 95% and 68% confidence intervals (CI) of the 5,000 filtered simulations are shown as thick line, and light and dark red shadings, respectively). ODP976 ${{\rm{U}}}_{{\rm{E}}}^{{\rm{K}}^{\prime} }$ -temperature reconstruction is also shown in blue^2,29^. Light and dark blue shadings in (**c**) correspond to the 95% and 68% CI of 5,000 filtered Monte Carlo simulations of the ${{\rm{U}}}_{{\rm{E}}}^{{\rm{K}}^{\prime} }$-temperature and ages within their (1σ) uncertainties. We also show the 95% CI of the 5,000 filtered simulations of the *Δ*_47_-replicates in (**b**). Red stars show ODP975 *δ*^18^O_C_ (**a**) and *Δ*_47_-temperature (**c**) late-Holocene values. Gaps in the final *Δ*_47_-record correspond to intervals where the age uncertainties of the *Δ*_47_-replicates do not overlap.

We first present the new paired records of *δ*^18^O_C_ and *Δ*_47_-temperature in the wMed, using Ocean Drilling Program (ODP) Site 975 (ODP975, Fig. 1). We use the *Δ*_47_-temperature calibration from ref.^19^, which was established with the same equipment used here (Methods) and which has been recently confirmed by other laboratories^20^. Our ODP975 paired *Δ*_47_-*δ*^18^O_C_ measurements (Fig. 2a,b) use the winter/spring surface-dwelling foraminifer *Globigerina bulloides*^4^. Initially, we generate a *Δ*_47_-temperature record using the traditional binning method that calculates weighted averages for ~10 *Δ*_47_-replicates that are clustered *a-priori* on the basis that they come from the same or close-neighbour (up to 1 kyr apart) samples (Methods). This gives a *Δ*_47_-temperature record (Fig. 2c, dots) of very low resolution, which nevertheless broadly depicts cooling associated with HS11 and subsequent warming into the last interglacial period. Results corroborate the reliability of the *Δ*_47_-small^17,25,26^ method in detecting glacial-interglacial temperature offsets.

For a more detailed assessment of the amplitude and timing of the events, we then apply our new, more thorough analysis of the *Δ*_47_-data (see Supplementary information for a detailed explanation and validation of this approach). In summary, we perform an outlier test on all samples with ≥5 *Δ*_47_-replicates. Then, we execute 5,000 Monte Carlo simulations of all the *Δ*_47_-replicates that remain after outlier removal. Each simulated dataset was converted into *Δ*_47_-temperature^19^, and a ~5 kyr moving Gaussian window (1σ = 0.8 kyr), stepping in 0.1 kyr increments, was applied to each simulation to highlight the main trends. By the end of these steps we have a total of 5,000 filtered *Δ*_47_-temperature simulations. The 50^th^ percentile (median), and the 16^th^–84^th^ and 2.5^th^–97.5^th^ percentiles of these filtered simulations form our final record, 68% Confidence Interval (CI) and 95% CI, respectively (hereafter, results are discussed using the 68% CI). The resultant ODP975 *Δ*_47_-record documents a cooling step of ~6 ± 2 °C at ~135–130 ka, followed by a warming of 12 ± 3 °C. This is in agreement (within uncertainties) with the ${{\rm{U}}}_{37}^{{\rm{K}}^{\prime} }$-temperature record from nearby ODP Site 976 (ODP976 ref.^29^), which has been previously synchronized with ODP975 (ref.^2^). This agreement is further emphasized when both reconstructions are normalized to the late-Holocene temperature value (Supplementary Fig. 1a), which eliminates biases related to different ecological preferences of the signal-carriers (e.g., depth habitat).

Combined with a late Holocene *Δ*_47_-temperature of 17 ± 2 °C (Fig. 2c), and its similarity to today’s spring/winter (~18/15 °C) temperatures^31^ at ODP975, the agreement with the nearby ODP976 ${{\rm{U}}}_{37}^{{\rm{K}}^{\prime} }$-record (Fig. 2c and Supplementary Fig. 1a) instils confidence in the *Δ*_47_-small method^25^, calibration^19^, and non-traditional data-analysis approach (Methods). This agreement is especially obvious in intervals of ODP975 where closely spaced *Δ*_47_-replicates were available (notably, 140–129 ka, Supplementary Fig. 1a) because this facilitates outlier detection and allows significant estimated error reduction (Supplementary Fig. 2a–c). Intervals where *Δ*_47_-replicates are relatively scarce result in larger uncertainties (e.g., 125–122 ka, Supplementary Fig. 2a–c), but remain consistent with the ODP976 ${{\rm{U}}}_{37}^{{\rm{K}}^{\prime} }$-record (Supplementary Fig. 1a). Intervals where no *Δ*_47_-replicates were available due to near-absence of *G*. *bulloides*, and the age uncertainties of neighbour samples do not overlap (e.g., 128–126 ka and ~118.5 ka in Fig. 2b), were removed from the final records to avoid overinterpretation of *Δ*_47_-interpolations across these gaps. Overall, we infer that the *Δ*_47_-method and data treatment used here delivers reliable records of temperature change at palaeoclimatically useful resolutions, especially as the density of *Δ*_47_-replicates increases.

### Paired *Δ*_47_-*δ*^18^O_C_ measurements in the eastern Mediterranean Sea

We applied the same procedure to two eMed cores, where use of traditional foraminifer-based geochemical methods is challenging^12,13^. Also, eMed ${{\rm{U}}}_{37}^{{\rm{K}}^{\prime} }$-based reconstructions^32,33^ are of limited use for comparing with our *Δ*_47_-based results because eMed bottom waters switched from highly oxygenated to anoxic conditions across the studied interval^7,32^, which affected alkenone preservation and thus the reliability of ${{\rm{U}}}_{37}^{{\rm{K}}^{\prime} }$-based reconstructions^9^. Hence, in the eMed we investigate whether *Δ*_47_-based reconstructions are spatially replicable, using the summer mixed-layer dwelling^4^ planktic foraminifer *Globigerinoides ruber* (white) in cores LC21 and ODP967 (Fig. 1), where similar climatic forcing conditions prevail.

**Figure 3:**
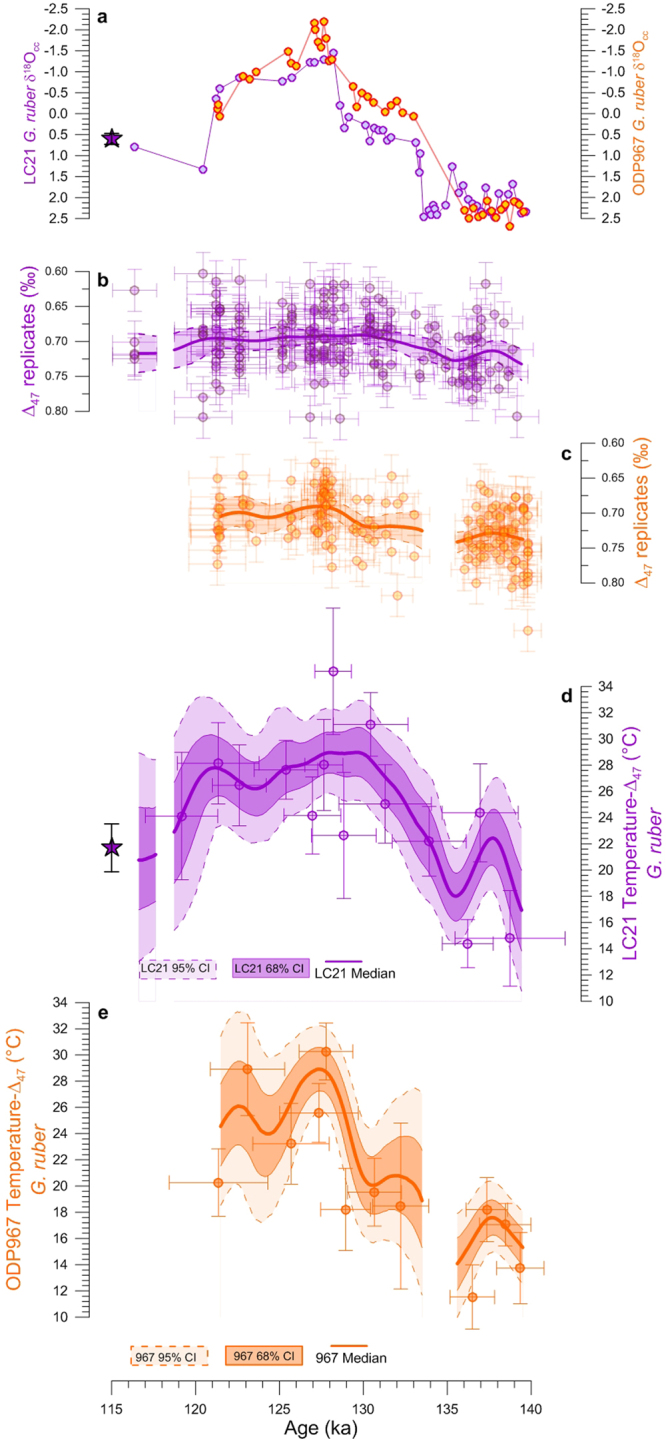
Eastern Mediterranean temperature records across TII. LC21 (purple) and ODP967 (orange) *G*. *ruber* (**a**) *δ*^18^O_C_ and (**b**,**c**) *Δ*_47_-replicates (shadings correspond to the 95% CI of the 5,000 filtered simulations of the *Δ*_47_-replicates). *G*. *ruber Δ*_47_-temperature records from (**d**) LC21 and (**e**) ODP967. Dots, thick lines, and shadings in (**d**,**e**) as in Fig. 2c. We also show in (**a**,**d**) the LC21*δ*^18^O_C_ and *Δ*_47_- temperature late-Holocene values (purple star), respectively. Gaps in the final *Δ*_47_-records correspond to intervals where the age uncertainties of the *Δ*_47_-replicates do not overlap.

**Figure 4:**
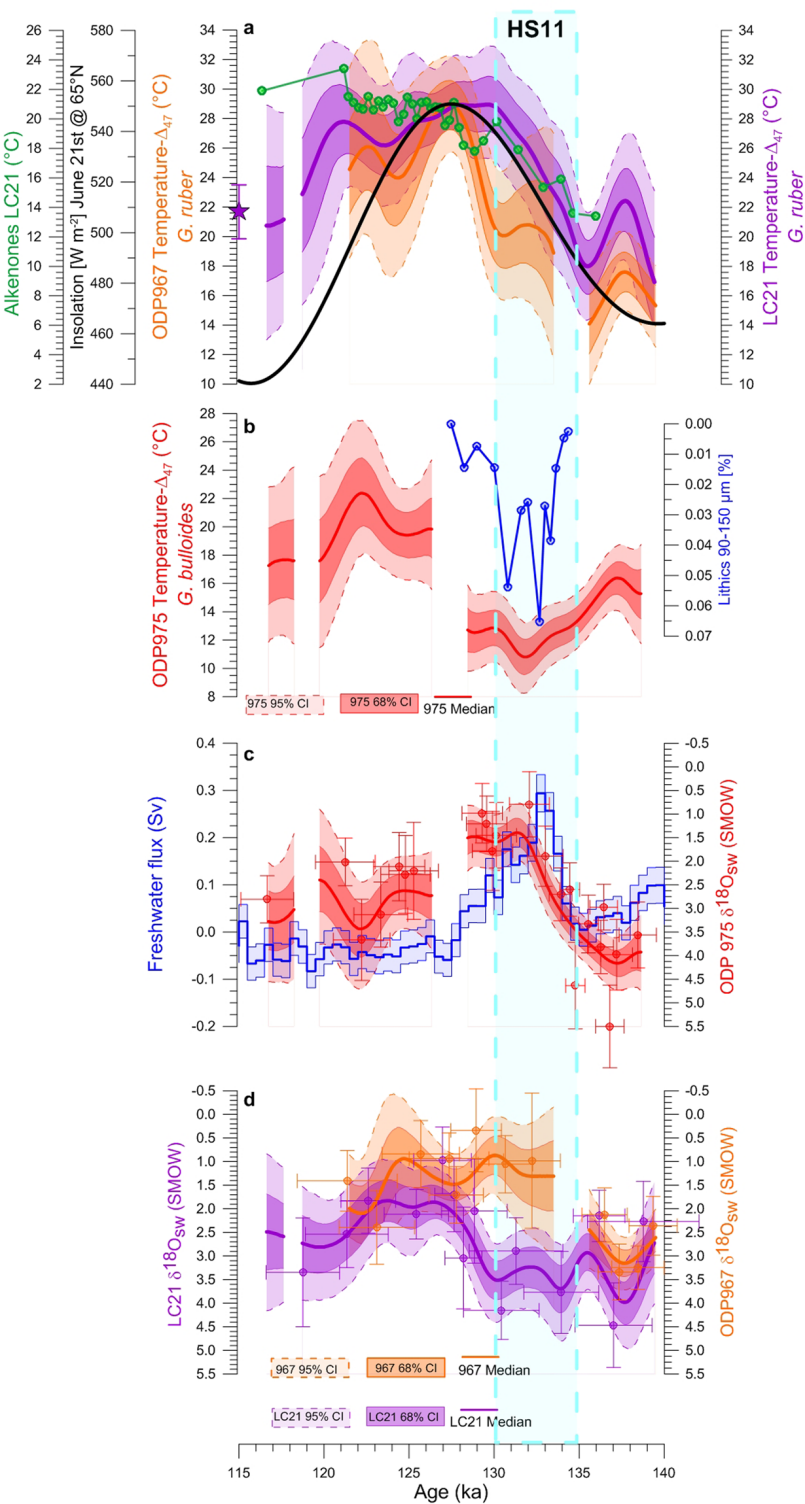
TII temperature and *δ*^18^O_SW_ records across the Mediterranean Sea. (**a**) Nothern Hemisphere insolation record at 65°N (black) overlain on the LC21 (purple) and ODP967 (orange) *Δ*_47_- temperature, and LC21 ${{\rm{U}}}_{{\rm{E}}}^{{\rm{K}}^{\prime} }$-temperature^32^ (using ref.^6^ age model) records from the eMed. (**b**) ODP975 *G*. *bulloides Δ*_47_-temperature record (red) and ice-rafted debris (Lithics 90–150 μm, %) concentrations from the Iberian Margin (blue^39^). (**c**) Freshwater fluxes into the North Atlantic (blue^2^) and ODP975 *G*. *bulloides*-*δ*^18^O_SW_ (red). (**d**) *G*. *ruber δ*^18^O_SW_ records from LC21 (purple) and ODP967 (orange). Dots, thick lines, and shadings as in Fig. 2c. LC21 *Δ*_47_ late-Holocene temperature (purple star). Gaps in the final *Δ*_47_-records correspond to intervals where the age uncertainties of the *Δ*_47_-replicates do not overlap. Blue box highlight the HS11.

Our eMed paired Δ_47_-δ^18^O_C_ measurements (Fig. 3a–c) show a gradual warming of 11 ± 3 °C and 15 ± 3 °C across TII at LC21 and ODP967 (Fig. 3d,e), respectively. At the LC21 site this warming is preceded by a brief ~4 ± 3 °C cooling at ~135 ka. We infer that this cooling is also evident in the ODP967 record, though its upward leg is not resolved because of a short gap at ~135–133ka (Fig. 3c and e). In general, the two eMed *Δ*_47_-records are consistent at the 95% CI (Fig. 4a). At 68% CI, however, local millennial-scale differences are observed (e.g., at ~130 ka), which result from an apparent slowdown of the warming trend at ODP967 across the interval of HS11. In contrast, the warming trend is more gradual and monotonic in the LC21 *Δ*_47_-record. A ${{\rm{U}}}_{37}^{{\rm{K}}^{\prime} }$-based reconstruction^32^ from LC21 supports this gradual and monotonic warming trend across HS11, despite low resolution owing to poor organic matter preservation prior to the S5 sapropel^30^ ~128–122 ka (Fig. 4a). Hence, it appears that the eMed record of ODP967 shows some vestiges of the HS11 cooling that is so prominent in the wMed (Fig. 4b), while the eMed record of LC21 shows no trace of it.

Next, we compared both eMed *Δ*_47_-records to the 22 ± 2 °C late Holocene *Δ*_47_-temperature average for LC21 (Fig. 3d), which is similar to modern 0–30 m depth averages at both core sites^31^ (~23 °C). This reveals that temperatures at LC21 during the penultimate glacial maximum were on average lower than late Holocene values by ~4 ± 2 °C, in agreement with previous estimates of the Last Glacial Maximum-to-Present temperature gradient^34^ (Supplementary Fig. 1b). A similar temperature gradient is inferred at ODP967 (Supplementary Fig. 1c). Last interglacial temperatures, on the other hand, are found to have been consistently higher than late-Holocene values at both core sites (Supplementary Fig. 1b,c).

### *δ*^18^O_SW_ records from the Mediterranean Sea through TII and the last interglacial period

Mediterranean *δ*^18^O_SW_ records derived from the paired *Δ*_47_-*δ*^18^O_C_ data are shown in Fig. 4c,d. The ODP975 *δ*^18^O_SW_ record (Fig. 4c) shows a ~2.8 ± 0.4‰ shift to lighter values from ~137 ka until ~128 ka, when surface-water *δ*^18^O_SW_ progressively returned to values almost as high as the glacial ones (~3.4 ± 0.5‰). We infer a similarly large change towards lower values (~1.8 ± 0.6‰) at the beginning of TII for the OD967 *δ*^18^O_SW_ record (Fig. 4d). However, at OD967 low surface *δ*^18^O_SW_ conditions persisted after ~128 ka. In contrast, the LC21 *δ*^18^O_SW_ record (Fig. 4d) only documents a muted ~1 ± 0.6‰ and transient (~2 kyr) change toward lower values at the beginning of TII. The most prominent shift of *δ*^18^O_SW_ to lower values (~1.6 ± 0.5‰) at this site is observed from ~128 to ~126 ka, and generally low values then persist until ~122 ka.

## Discussion

### Hydrographic changes across the Mediterranean Sea

Our results offer a first quantification of west-to-east hydrographic developments in the Mediterranean Sea through a deglaciation. Focusing on temperature first, our *Δ*_47_-temperature record based on the summer mixed-layer species *G*. *ruber* for eMed site LC21 reveals a gradual, monotonic warming through TII that broadly follows the Northern Hemisphere insolation from 136 ka (Fig. 4a). This pattern is consistent with the globally widespread pattern of temperature change through TII as observed in Antarctica^35^, the Indo-Pacific warm pool^36,37^, and in the Arabian Sea^38^. TII temperature changes in the wMed, on the other hand, clearly show more similarity to those seen in the North Atlantic, with a notable (6 ± 2 °C) cooling at ~135–130 ka (Figs 2c and 4b). This temperature drop coincides with ice-rafted debris deposition at the Iberian Margin and within the wider North Atlantic^39^ (Fig. 4b), and with large meltwater fluxes into the North Atlantic (Fig. 4c), which together signal the occurrence of HS11 (ref.^2^).

At eMed Site ODP967, temperature changes through TII are slightly more complex. While the ODP967 *Δ*_47_-temperature record is broadly consistent with that from LC21 at the 95% confidence level, its warming from 136 ka onward is not gradual and monotonic as in LC21, but contains a plateau between 133 and 130 ka that delays the final warming into the interglacial, relative to LC21 (Fig. 4a). This plateau at ODP967 may be a muted expression of the HS11 cooling, which is completely absent at LC21. Because of the plateau, the delayed (~130–128 ka) warming into the interglacial in ODP967 shows a closer timing similarity with temperature records from the wMed than LC21; this final warming in ODP967 leads that in the wMed by ~1 kyr while LC21 leads the wMed by about ~3 kyr (Supplementary Figs 1c and 3a). From ~128 ka, when sapropel conditions developed, sea surface temperatures in the eMed became more homogeneous (Fig. 4a).

Overall, we reveal that Mediterranean responses to climate forcing during TII display considerable spatial complexity, especially between the wMed and eMed, but also (albeit not distinguishable within 95% confidence limits) within the eMed. During HS11, the wMed record shows considerably stronger cooling than the eMed records (Fig. 4a,b, Supplementary Figs 1a–c and 3a). This may (partly) reflect the fact that we had to rely on a species that predominantly records winter/spring conditions for the wMed, whereas we had to rely on a species more typical of summer conditions for the eMed^4^. Thus, we infer with confidence that there were, at least, strong seasonal contrasts in the expression of HS11 cooling within the Mediterranean, and more tentatively that there may have been a remarkable attenuation of HS11 cooling from the wMed to the eMed. If validated by further analyses, the latter gradient would imply that strong HS11 cooling in the wMed reflects dominant influences of the adjacent North Atlantic conditions, while weaker HS11 impacts in the eMed are reminiscent of the overall, underlying, insolation-driven pattern that is also seen in the relatively nearby (sub-)tropical Arabian Sea^38^.

The *δ*^18^O_SW_ changes across the Mediterranean Sea also show considerable complexity (Fig. 4c,d). We infer that these records reflect a diverse response of the Mediterranean Sea to two sources (with distinctly different isotopic compositions) of freshwater input into the basin. One is from the North Atlantic at ~135–130 ka during HS11 (ref.^2^), and the other from the North African Margin^32^ at ~128.3–121.5 ka during the time of the Sapropel 5 (S5) deposition^30^. HS11 freshwater inputs from deglacial ice-sheet melting^2^ entered the Mediterranean Sea via the Strait of Gibraltar, reaching ODP975 where the *δ*^18^O_SW_ record documents the largest change (~2.8 ± 0.4‰) of all the reconstructions (Fig. 4c,d). The *δ*^18^O_SW_ data suggest that this freshwater anomaly progressively vanished along the path toward the eastern side of the basin, due to increasing evaporation and dense water formation close to the LC21 location^7^. This site is close to the Rhodes Gyre where intermediate water is formed^7^, which dilutes surface *δ*^18^O_SW_ signals that are advected to the region over a 4 to 5 times more voluminous system. This trajectory resulted in reduced and almost absent *δ*^18^O_SW_ anomalies at the sites of ODP967 and LC21 at ~135–130 ka, respectively. Interestingly, while their *δ*^18^O_SW_ records are different (Fig. 4d), the *Δ*_47_-based temperature developments at ODP967 and LC21 are broadly similar, within uncertainties (Fig. 4a, Supplementary Fig. 3a,b). This is because general temperature signals can be rapidly adjusted over large regions through atmospheric conditions, whereas changes in *δ*^18^O_SW_ rely on mixing of large regional gradients associated with particular freshwater fluxes. In more detail, more cool and low-*δ*^18^O_SW_ water appears to have reached ODP967 than LC21 in the eMed during HS11, which suggests a sharp regional gradient between ODP967 and LC21 in the advection of surface waters from the wMed, or attenuation of the anomalies due to stronger mixing with ambient waters at LC21 than at ODP967. After the HS11 interval, the North African Monsoon intensified as Northern Hemisphere insolation peaked and the Intertropical Convergence Zone moved northward^40,41^. This delivered large amounts of freshwater into the eMed around ~128–122 ka, pre-conditioning the basin, through water-column stratification^32,42^, for organic-rich (sapropel) sediment deposition^7^. This is evidenced in our *δ*^18^O_SW_ records by persistent fresh (low-*δ*^18^O_SW_) surface water conditions at ODP967 after HS11, along with initiation of surface freshening at the location of the LC21 also at ~128 ka (Fig. 4d).

### Last interglacial surface warming across the Mediterranean Sea

Previous work has suggested that the increased discharge of monsoon-fueled freshwater into the eMed during the last interglacial period may have amplified surface warming by leading to a strongly stratified upper water column^33^. Our data show that surface temperatures in the eMed during the last interglacial were consistently higher than late-Holocene temperatures, by up to ~7 ± 4 °C (95% CI) (Supplementary Fig. 1b,c). This is especially evident at ~128 ka, at the onset of S5 (ref.^30^). Although the last interglacial was globally warmer than the Holocene, the warming we observe in the Mediterranean Sea is much larger than that in global averages^43^. A ~30% thinning of the summer mixed layer at the S5 onset (Supplementary Table 1), concentrating the radiative energy gain, provides a more likely mechanism to explain the ~7 ± 4 °C (95% CI) excess warming. Thus, our evidence supports the theory that the *δ*^18^O_C_ anomaly in *G*. *ruber* across sapropels only partly reflects freshwater-induced *δ*^18^Osw changes, and for another part reflects anomalous warming of the restricted surface-water layer they inhabited^33^.

Our paired *Δ*_47_-*δ*^18^O_C_ data provide the first insights into the eMed hydrographic responses to a prominent deglacial Heinrich stadial. It also provides novel observational support to the importance of temperature changes in the development of water-column stratification during sapropel formation^33^. We conclude that our *Δ*_47_ approach has potential to solve other climate-related questions that have remained elusive because of shortcomings in traditional temperature proxies, notably when non-traditional data-analysis is combined with ongoing analytical advances^23^.

## Methods

### Sediment core locations and age models

We used samples from wMed Ocean Drilling Program (ODP) Site 975 (ODP975, 38°53.8′N, 4°30.6′E, 2,415 m water depth), eMed core LC21 (Aegean Sea, 35°40′N, 26°35′E, 1,522 m water depth) and eMed ODP967 (Eratosthenes Seamount, 34°04′N, 32°43′E, 2,553 m water depth). The chronology of LC21 is radiometrically constrained^6^. The age model of ODP975 has previously been related to that of LC21 (ref.^2^). Here we add synchronization of the ODP967 record to LC21 (Supplementary Table 2), based on: (*i*) Scanning X-ray fluorescence (XRF) Barium (Ba) (Supplementary Fig. 4a) to identify and tie the Sapropel S5 interval^30,44^ (128.3–121.5 ka) of the two sediment cores; and (*ii*) the ODP967 and LC21 *δ*^18^O records (Supplementary Fig. 4b). It is common practice to use foraminiferal *δ*^18^O records to synchronize sediment cores, but this was not possible in certain intervals for the ODP967 to LC21 synchronization, since ODP967 contains two intervals (B2H1 137–143 cm and B2H2 6–13 cm) across TII where foraminiferal numbers drop to very low values. To overcome this, we took advantage of similarities in the bulk-sediment *δ*^18^O records (see measurement details below) between LC21 and ODP967. The ODP967 age model includes (*iii*) an age of 175.63 ka which corresponds to the midpoint^44^ of Sapropel S6 in ODP967. The synchronization between LC21 and ODP967 was validated using the high resolution foraminiferal *δ*^18^O_C_ and carbon isotope (*δ*^13^C_C_) records of LC21 (ref.^6^) and ODP967 (Supplementary Fig. 4c,d). Changes in the *δ*^18^O_C_ and *δ*^13^C_C_ records from LC21 and ODP967 coincide, especially in the S5 interval. Further confidence in the OPD967 age model is given by the synchronous occurrence of a cooling step at ~135 ka in both *Δ*_47_*-*temperature reconstructions (Fig. 3d,e). Overall, synchronisation uncertainties between the three cores are smaller than a thousand years and thus do not affect the conclusions presented in our study.

### Sample preparation

For ODP975, we picked *Globigerina bulloides* from the size fraction 300–355 μm to obtain ~1.5–2 mg of carbonate. We commonly use a narrow size window to minimize size-dependent vital effects. However, in some cases *G*. *bulloides* was so rare (e.g., last interglacial period) that we had to pick from a wider size window (250–355 μm). For LC21 and ODP967, we picked *Globigerinoides ruber* (white). In LC21, its abundances were very low within the glacial and at the beginning of TII, which is reflected in the low number of *Δ*_47_-replicates through this interval (~140–132 ka, Fig. 3b), and the wide specimen size window that we had to use (300–450 μm, or wider). In ODP967, we could be more rigorous with the size window (300–400 μm), as *G*. *ruber* (w) was reasonably abundant throughout the study interval, except in the two gaps at around ~136–133 ka and ~129 ka.

Prior to analysis, picked foraminifera were crushed. For ODP975 and LC21, samples were cleaned as follows: we added methanol to the vial and sonicated the sample for ~10 sec. We then waited a few seconds until the carbonate sample settled in the bottom of the vial and removed the supernatant solution. This procedure was repeated until the sample was visually clean, i.e., the milky aspect of the supernatant dissapeared. Samples were dried in an oven at 45 °C for less than 12 hr. ODP967 samples required further cleaning, using the full Mg/Ca clay-removal step outlined in ref.^45^, which uses several rinses with both water and methanol.

### Bulk sediment *δ*^18^O analytical procedure

Prior to analyses, LC21 and ODP967 sediment samples were ground. LC21 samples were analyzed at the National Oceanography Centre, Southampton using a Europa Geo2020 mass spectrometer following same procedures as in ref.^6^. ODP967 samples were analyzed at the Australian National University using a Thermo Fisher Scientific Delta Advantage mass spectrometer coupled to a Kiel IV carbonate device for sample digestion. Isotope data were normalized to the Vienna Peedee Belemnite (VPDB) scale using NBS-19. External reproducibility (1σ) was always better than 0.08‰.

### Foraminiferal *δ*^18^O and *Δ*_47_ analytical procedure

The clumped isotope thermometer is defined as the excess of CO_2_ isotopologue with mass 47 (^13^C-^18^O-^16^O) in a sample gas with respect to the expected abundance if the isotopes were randomly distributed among all isotopologues^14,15,46^. It is reported as *Δ*_47_:

1 $${\Delta }_{47}=[(\frac{{{\rm{R}}}^{47}}{{{\rm{R}}}^{47\ast }}-1)-(\frac{{{\rm{R}}}^{46}}{{{\rm{R}}}^{46\ast }}-1)-(\frac{{{\rm{R}}}^{45}}{{{\rm{R}}}^{45\ast }}-1)]\times 1000$$

where R^47^, R^46^, and R^45^ correspond to the relative abundance of the cardinal masses 47, 46, and 45 with respect to the mass 44 in the sample. R^47^*, R^46^* and R^45^* are the expected ratios in the sample if it had a stochastic distribution among all isotopologues, which are calculated from the measured *δ*¹³C and *δ*^18^O (refs^21,47^).

We followed the procedure for obtaining the isotopic composition (*δ*^13^C_C_ and *δ*^18^O_C_) and state of ordering (*Δ*_47_) in small carbonate samples as reported in ref.^25^. Briefly, samples were analysed using a Thermo Fisher Scientific MAT 253 mass spectrometer coupled to a Kiel IV automated carbonate preparation device, where each sample is reacted with phosphoric acid at 70 °C. The Kiel IV device has a PoraPak Q trap^26^ to retain organic contaminants that could bias the *Δ*_47_ towards high values. Prior to analysis of each sample run, the pressure-dependent backgrounds were determined on all beams to correct for non-linearity effects of the mass spectrometer according to ref.^48^. During each run, at least 18 replicates of different samples and 6 replicates of each of the carbonate standards ETH-1, ETH-2, ETH-3 and ETH-4 were analysed. The *δ*^13^C_C_, *δ*^18^O_C_, and *Δ*_47_ (in the Absolute Reference Frame or carbonate dioxide equilibrated scale^49^, CDES) for the standards are listed in Supplementary Table 3. For most of the ODP975 and LC21 samples each analysis consisted of 8 cycles of sample and reference gas comparisons (26 s) interrupted by 10 s of idle time. For ODP967 samples we used the Long-Integration Dual-Inlet (LIDI) method^24^. During LIDI analysis, the sample gas was measured continuously for ~600 s, and then the reference gas for the same time starting at the same signal intensity on *m/z* 44 as the sample gas. The raw *Δ*_47_ was calculated by matching reference and sample gas beam intensities after the measurement. The reproducibility of each *Δ*_47_ -replicate is ~0.03‰ (1σ).

During data processing, standards with equal (different) *Δ*_47_ and different (equal) *δ*^47^ were used to check the PBL correction (scale compression) as well as offsets during the measurement^25^. The *Δ*_47_ for each sample was calculated using its bulk isotopic composition as described in ref.^21^ after applying the pressure-dependent background correction^48^. Then the data were corrected for scale compression by anchoring the values to the Absolute Reference Frame^49^ using the carbonate standards, and corrected for the specific acid digestion temperature^50^. Data processing for ODP967 was done with the recently developed Easotope software^51^. *δ*^13^C and *δ*^18^O raw data were normalized to NBS-19 VPDB scale using the ETH-1 and ETH-2 standards. During LIDI analysis, a total of 18 *Δ*_47_-replicates of a Marula Marble standard (ANU-M2) were measured along with the samples, distributed throughout the measuring period. They yielded average values of *δ*^13^C = 2.80 ± 0.03‰ (1σ), and *δ*^18^O = −7.33 ± 0.09‰ (1σ) consistent with the ANU values for the standard (*δ*^13^C = 2.84 ± 0.04‰ (1σ) and *δ*^18^O = −7.4 ± 0.1‰ (1σ). The ANU-M2 *Δ*_47_ value was 0.407 ± 0.02‰ (1σ) based on 18 *Δ*_47_-replicates, which indicates a good external reproducibility for the period of the analyses. The ANU-M2 *∆*_47_ average value is equivalent to a temperature of 194 ± 6 °C when using ref.^19^, which is very close to the value found for other marbles such as NBS19 and other Carrara marbles^52^.

### Temperature reconstructions using the traditional *Δ*_47_-approach

The *Δ*_47_-small method uses ~10 *Δ*_47_-replicates to calculate the weighted *Δ*_47_-average of a sample^25,26^. In this study the *Δ*_47_-average was calculated in most of the cases by inclusion of *Δ*_47_-replicates from neighbouring samples (up to 1 kyr apart) and/or different size fractions. Note that these were always analysed separately, that is, never “homogenized” prior to analyses. We calculated the weighted average, based on the distance of each *Δ*_47_-replicate to the average age of those 10 (or more) *Δ*_47_-replicates, using a tricube function^53^. The age of the *Δ*_47_-average is the weighted average age of the *Δ*_47_-replicates and the associated age error was propagated using the root mean square errors method.

Each *Δ*_47_-average is reported with the associated standard error (SE).

2 $$SE=\frac{\sigma }{\sqrt{N}}$$

where *σ* corresponds to the standard deviation of the 10 (or more) *Δ*_47_-replicates, and *N* is the number of *Δ*_47_-replicates used to calculate the *Δ*_47_-average. The *Δ*_47_-average was converted to temperature using the *Δ*_47_-Temperature calibration^19^ (equation 3), and we used the root mean square errors method to propagate the analytical uncertainties.

3 $${\Delta }_{47}=\frac{(0.044\pm 0.005)\ast {10}^{6}}{{T}^{2}}+(0.205\pm 0.0047)$$

where *Δ*_47_ is reported in per mil and T is the absolute temperature.

### Temperature and *δ*^18^O_SW_ calculations using the non-traditional data-analysis approach

Here, uncertainties in the independent (Age) and dependent (e.g., *Δ*_47_, temperature and *δ*^18^O) variables were propagated using 5,000 Monte Carlo simulations of the datasets, previously screened for outliers. Then, a 5 kyr Moving Gaussian Window filter (MGW) was applied to each simulation, shifting in 100 year increments. The final *Δ*_47_, temperature, and *δ*^18^O_SW_ records are shown as the median (50^th^ percentile) of the 5,000 MGW filtered simulations of each dataset. The 95% and 68% CI of each record are given by the 2.5^th^–97.5^th^ and 16^th^–84^th^ percentiles of the 5,000 MGW filtered simulations, respectively. To deconvolve the *δ*^18^O_SW_ we used refs^54,55^ for *G*. *bulloides* in the wMed record. For the eMed records we used the Temperature-*δ*^18^O_c_ relationship for *Orbulina universa* in ref.^54^, which is commonly used for *G*.*ruber*^56^. See Supplementary information for a more detail explanation of this approach.

## Electronic supplementary material

Supplementary Information
