# Author Correction: Penultimate deglacial warming across the Mediterranean Sea revealed by clumped isotopes in foraminifera

**DOI:** 10.1038/s41598-021-96895-3

**Published:** 2021-08-26

**Authors:** L. Rodríguez-Sanz, S. M. Bernasconi, G. Marino, D. Heslop, I. A. Müller, A. Fernandez, K. M. Grant, E. J. Rohling

**Affiliations:** 1 Research School of Earth Sciences, The Australian National University, Canberra, Australian Capital Territory 2601 Australia; 2 Geological Institute, ETH Zurich, Sonneggstr. 5, 8092 Zurich, Switzerland; 3 Present Address: University of Vigo, Campus Universitario, 36310 Vigo, Spain; 4 Ocean and Earth Science, University of Southampton, National Oceanography Centre, Southampton, S014 3ZH UK

Correction to: *Scientific Reports* 10.1038/s41598-017-16528-6, published online 29 November 2017

This Article contains errors in Figures 3 and 4.

In Figures 3a (orange), 3e and 4c-d (red and orange) the filled circles were incorrectly plotted.

The correct Figures 3 and 4 and accompanying legends appear below.

**Figure 3:**
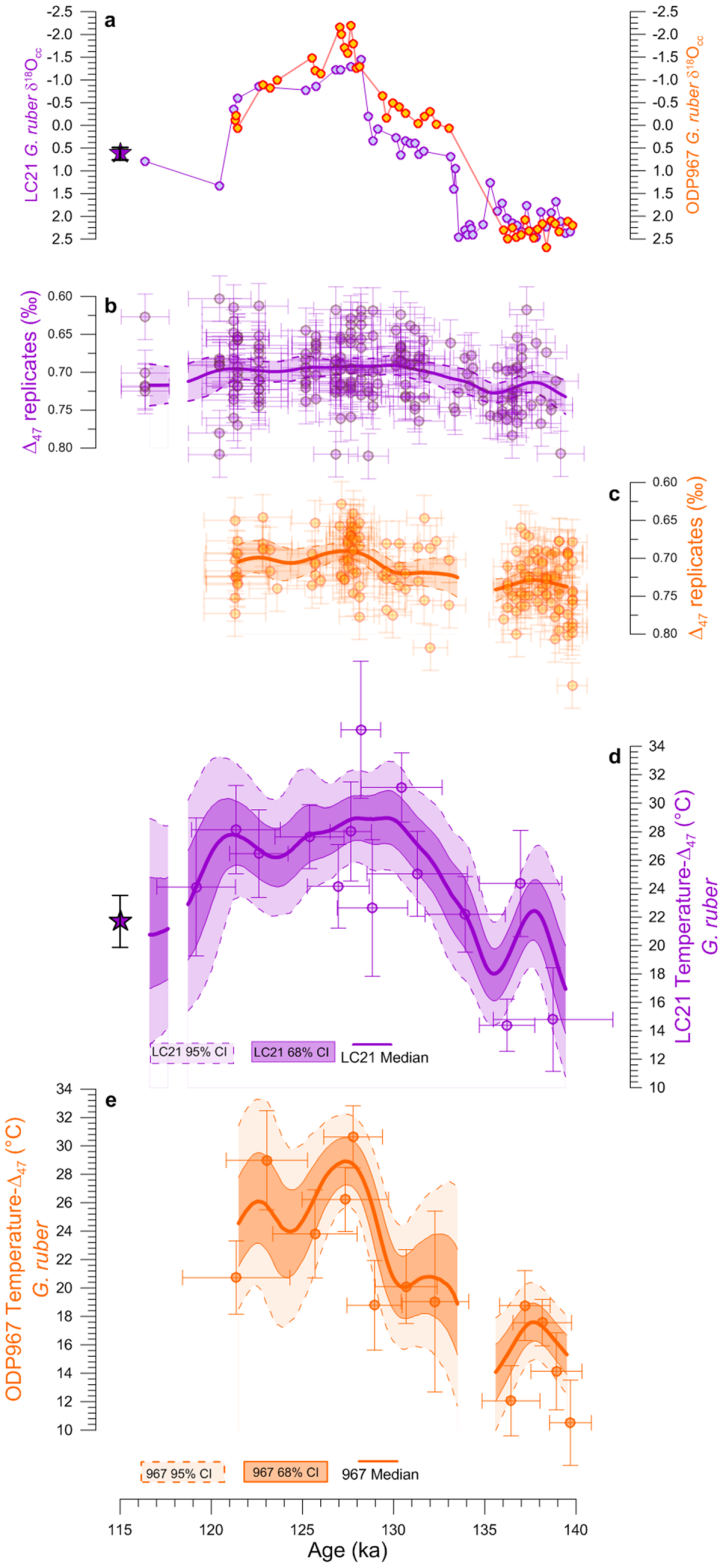
Eastern Mediterranean temperature records across TII. LC21 (purple) and ODP967 (orange) *G*. *ruber* (**a**) *δ*^18^O_C_ and (**b**,**c**) *Δ*_47_-replicates (shadings correspond to the 95% CI of the 5,000 filtered simulations of the *Δ*_47_-replicates). *G*. *ruber Δ*_47_-temperature records from (**d**) LC21 and (**e**) ODP967. Dots, thick lines, and shadings in (**d**,**e**) as in Fig. 2c. We also show in (**a**,**d**) the LC21 *δ*^18^O_C_ and *Δ*_47_-temperature late-Holocene values (purple star), respectively. Gaps in the final *Δ*_47_-records correspond to intervals where the age uncertainties of the *Δ*_47_-replicates do not overlap.

**Figure 4:**
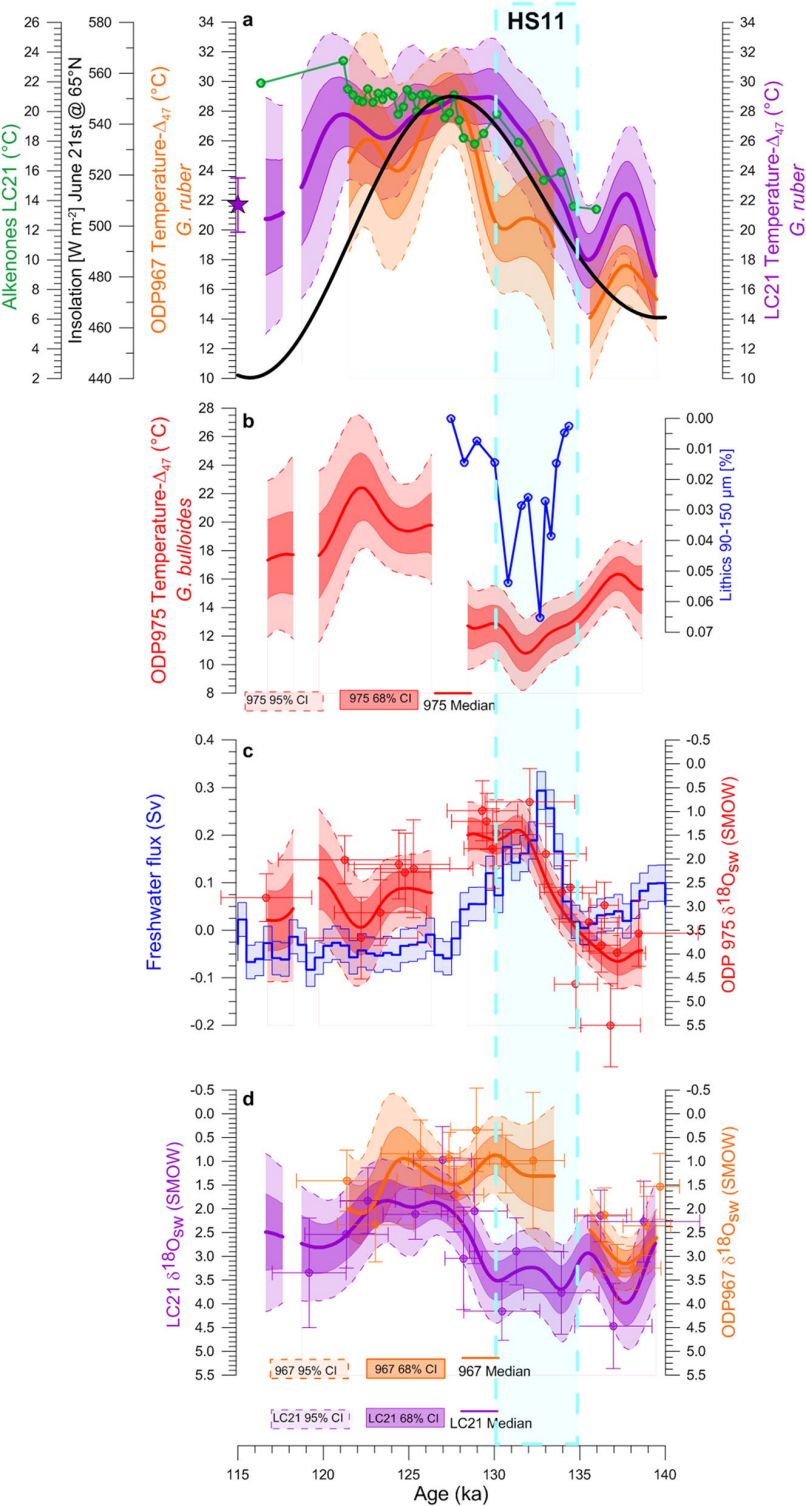
TII temperature and *δ*^18^O_SW_ records across the Mediterranean Sea**.** (**a**) Northern Hemisphere insolation record at 65°N (black) overlain on the LC21 (purple) and ODP967 (orange) *Δ*_47_-temperature, and LC21 $\text{U}_{37}^{\text{K}^{\prime}}$-temperature^32^ (using ref.^6^ age model) records from the eMed. (**b**) ODP975 *G*. *bulloides Δ*_47_-temperature record (red) and ice-rafted debris (Lithics 90–150 μm, %) concentrations from the Iberian Margin (blue^39^). (**c**) Freshwater fluxes into the North Atlantic (blue^2^) and ODP975 *G*. *bulloides*-*δ*^18^O_SW_ (red). (**d**) *G*. *ruber δ*^18^O_SW_ records from LC21 (purple) and ODP967 (orange). Dots, thick lines, and shadings as in Fig. 2c. LC21 *Δ*_47_ late-Holocene temperature (purple star). Gaps in the final *Δ*_47_-records correspond to intervals where the age uncertainties of the *Δ*_47_-replicates do not overlap. Blue box highlights the HS11.

