# Supplementary material for: Penultimate deglacial warming across the Mediterranean Sea revealed by clumped isotopes in foraminifera
Source: Sci Rep. 2017 Nov 29;7:16572. doi: 10.1038/s41598-017-16528-6 (PMC5707372; doi:10.1038/s41598-017-16528-6)
Supplement: Supplementary file 1 — Supplementary Information [file 41598_2017_16528_MOESM1_ESM.pdf]

Penultimate deglacial warming across the Mediterranean Sea revealed by clumped isotope in foraminifera

L. Rodríguez-Sanz[1*], S. M. Bernasconi[2], G. Marino[1], D. Heslop[1], I. A. Müller[2], A. Fernandez[2], K. M. Grant[1] & E.J. Rohling[1,3]

[1]Research School of Earth Sciences, The Australian National University, Canberra, Australian Capital Territory 2601, Australia.

[2]Geological Institute, ETH Zurich, Sonneggstr. 5, 8092 Zurich, Switzerland.

[3]Ocean and Earth Science, University of Southampton, National Oceanography Centre, Southampton S014 3ZH, UK

[*]Correspondence to laura.rodriguez@anu.edu.au

SUPPLEMENTARY INFORMATION

Supplementary information

Supplementary Figures 1-4 are called in the main text and methods to further clarify and support our conclusions. Supplementary Figures 5-11 are discussed in the text below.

Validation of the non-traditional Δ_{47} data-analysis approach

Data smoothing. The Δ_{47}-based time series is a mixed signal between the ambient seawater temperature recorded in the foraminiferal calcite, instrument shot-noise limit, measurement error, sample preparation artefacts, and other unidentified sources of uncertainty[1]. In order to reduce the high variability that these inflict on the Δ_{47}-data and highlight the main trends, we apply a Moving Gaussian Window (MGW) filter. We use this smoothing method because it allocates weights for averaging the Δ_{47}-replicates based on the size of the Gaussian window. This allows one of the key advancements of our data processing approach, i.e., it does not rely on any *a-priori* knowledge to obtain the final Δ_{47}-temperature records, as opposed to the traditional binning method. That is, for the traditional Δ_{47}-small method it is necessary to define the boundaries for averaging Δ_{47}-$\delta^{18}O_C$ replicates (binning) from neighbour samples. Unfortunately, that requires *a-priori* information that is not always available. Although the simultaneously generated and higher-temporal-resolution $\delta^{18}O_C$ record can be used to define the binning boundaries, this relies on the assumption that temperature and $\delta^{18}O_C$ co-vary, which is not always the case. In the wMed, for instance, temperature and $\delta^{18}O_C$ records across HS11 are markedly asynchronous[2,3] (Fig. 4b-c). The approach we propose in this study only requires defining the size of the MGW, which here is referred to in terms of the 99.7 % coverage ($\pm3\sigma$) of a Gaussian distribution in kyr. In general, the larger the size of the window, the smaller the estimated error of the reconstructed parameter, and the smoother the record. In our case we use a ~5 kyr window ($\pm1\sigma$ = 0.8 kyr) because it provides a reasonable compromise between associated uncertainties and the length of the climate events we are investigating. Additionally, the

frequency response of the 5 kyr MGW filter applied to an evenly spaced white noise signal that spans the same time interval of our data (Supplementary Fig. 5) shows that it satisfies the goal of reducing the high frequencies in the record. That is, the 5 kyr MGW should minimize the noise and highlight the main trend of the Δ_{47}-data.

Outlier test. The outlier test is based on the box-plot approach, which is an intuitive and broadly recommended method for data analysis, e.g., in medical research[4,5]. It has the advantage of detecting outliers regardless of the distribution of the sample and for this reason we implemented it in the approach. The box-plot uses the 25th (Q1), 50th (Q2), and 75th (Q3) percentiles of the data to characterize the sample (see ref. [4] for a detailed explanation). Data points (Δ_{47}-replicates) are considered outliers if they fall outside the Q3+W*(Q3-Q1) and Q1-W*(Q3-Q1) range. The standard approach of Tukey[6] uses W = 1.5. In the case of Gaussian distributed data, that value would identify Δ_{47}-replicates outside a $\pm 2.698\sigma$ range (99.3 % coverage) as outliers. We consider this range too large to detect outliers in our dataset and therefore we also tried a conservative definition that would consider outliers Δ_{47}-replicates falling outside a $\pm 2\sigma$ (~95.5 % coverage) range in a normal distribution (W = 0.9826). Supplementary Figure 6 shows that the final ODP975 record using W = 1.5 is not much different from those obtained using W = 0.9826 (main figures). The records only diverge where the density of the Δ_{47}-replicates is low at, e.g. 122-115 kyr in LC21. Yet, because (i) we focus our discussion on the interval between 140-122 kyr and (ii) the MGW filter is sensitive to outlying data, we favoured the conservative test, even if this implies that a few "potentially good" Δ_{47}-replicates might be wrongly removed from the dataset.

Monte Carlo simulations. We tested the effect of the number of Monte Carlo simulations in the final ODP975 Δ_{47}-temperature record. We run 4 different simulation ensembles: 500, 1,000,

5,000, and 10,000. We observe that, while there is no difference in the final Δ_{47}-temperature record, there is a slight effect on how smooth the confidence intervals appear. Fewer simulations result in a slightly less smoothed record (Supplementary Fig. 7). We selected N = 5,000 for our study because it offers a good compromise between the smoothness of the confidence intervals and the time that the simulations take.

Details on the non-traditional data-analysis MATLAB script. First, three files have to be provided in the *DATA INPUT* section: *data, Age_Model, and Sea_Level*. *Data* contains individual measurements of Δ_{47} (‰, CDES) and $\delta^{18}O$ (‰, VPDB) with its associated depth (in cm). *Age_Model* has the tie points (i.e., depth (cm), age (ka BP), ±1σ error age (kyr) for the age model. *Sea_Level* has the SL reconstruction[7] that will be used to correct for the ice volume component of the $\delta^{18}O$ record ($\delta^{18}O_{SW}$-ivc) using specific wMed and eMed $\delta^{18}O_{SW}$ -SL relationships[3]. The MATLAB script calculates the $\delta^{18}O_{SW\text{-}IVC}$ for future applications. For our discussion, there is no appreciable difference whether we use $\delta^{18}O_{SW}$ or $\delta^{18}O_{SW\text{-}IVC}$. Therefore, we used $\delta^{18}O_{SW}$ for simplicity. *DATA INPUT* section is followed by the *PARAMETERS* section where the number of Monte Carlo simulations, the size of the MGW, steps increments, and the coefficients for the equations that best fit the dataset have to be specified. Calculation steps are divided into three parts (see also Supplementary Fig. 8).

(i) ***Δ_{47}-replicates outlier test and Age model****:* Using the information in *Data* the outlier test is computed where more than 4 replicates (Δ_{47}-replicates>4) in the exact same sample were possible. The ages of the Δ_{47}-replicates deemed to not be outliers are then calculated using the tie points in *Age_Model*. Based on this, the MATLAB script generates a *dataset* containing the depth (cm), $\delta^{18}O$ (‰, VPDB), $\delta^{18}Oc$ ±1σ std (0.08 ‰), Δ_{47} (‰), Δ_{47} ±1σ std (0.03 ‰), age (kyr), and ±1σ error age (kyr), which is used afterwards for the Monte Carlo realizations.

(ii) ***Monte Carlo simulations*:** First, 5,000 realizations of Age based on its ±1σ chronological uncertainties are performed using a random walk Monte Carlo routine that employs a Metropolis–Hastings approach to reject steps in the random walk that will result in age reversals. Then, 5,000 realizations of the dependent variable (e.g., Δ_{47}, $\delta^{18}O$, and SL) are performed by generating normally distributed values of each data point based on their ±1σ uncertainties. For each realization, the following steps are carried out: **(a)** the Δ_{47}-data are converted into temperature using equation 3. **(b)** This temperature value is used to correct the temperature component of the $\delta^{18}O$ record and to thus determine $\delta^{18}O_{SW.}$ The latter is done by using realizations of the Temperature-$\delta^{18}O_c$ relationship based on its ±1σ uncertainty (specified in *PARAMETERS*). **(c)** The SL component of the derived $\delta^{18}O_{SW}$ is corrected for using realizations of the $\delta^{18}O_{SW}$-SL relationship[3] for the wMed (ODP975) or eMed (LC21, ODP967) (specified in *PARAMETERS*). **(d)** A 5 kyr MGW (size specified in *PARAMETERS*), stepping in increments of 100 years, is applied to each of the 5,000 times simulated datasets, i.e. Δ_{47}, Δ_{47}-temperature, $\delta^{18}O$, $\delta^{18}O_{SW}$, and $\delta^{18}O_{SW\text{-}ivc}$.

(iii) ***Final records and probability intervals:*** The 2.5th, 16th, 50th, 84th, and 97.5th percentiles of all the 5,000 MGW filtered Monte Carlo realizations of the Δ_{47}, Δ_{47}-temperature, $\delta^{18}O$, $\delta^{18}O_{SW}$, and $\delta^{18}O_{SW\text{-}IVC}$ datasets are calculated.

Efficiency of the approach in removing noise: The residuals of a model that adequately represents the data are *(a)* normally distributed, *(b)* independent (i.e., are not autocorrelated), and *(c)* are homoscedastic (i.e., have constant variance)[8]. We checked the efficiency of the non-traditional Δ_{47} data-analysis approach by analysing the residuals between the measured Δ_{47}-

replicates and the 50th percentile of the Δ_{47} MGW filtered Monte Carlo realizations (Supplementary Fig. 9-11).

(a) We checked that the residuals were normally distributed by applying the so-called 'Lilliefors Test'[9] at the 0.05 significance level. The histogram and Quantile-Quantile plot of the residuals are also shown, along with the residuals (Supplementary Fig. 9-11a-c), to visually corroborate the results of the Lilliefors test.

(b) We tested that the residuals were not auto-correlated by estimating their persistence time (τ) using a first-order autoregressive model (AR(1), ref. [10]). We expect τ to be small when the model residuals are independent. We first tried to estimate τ using the TAUEST software[10]. It returned an error because our records are not only unevenly spaced but also some ages are replicated. Hence, we extracted equations 2-3 from TAUEST (ref. [10]) to estimate if fluctuations in the residuals have a memory (if any) considerably smaller than the events of interest, e.g., τ<1000yrs. Equation 2 in ref. [10] defines the parameter a as a function of τ; $a = \exp(-1/\tau)$, and equation 3 in ref. [10] is the least-squares estimator, S(a), which is minimized to estimate a. We calculated S(a) for the Δ_{47} residuals 5,000 times using the unique ages (i.e., no replicated ages), with the option of randomly drawing a Δ_{47} residuals value when a sample has Δ_{47}-replicates >1. We compare the results to that of a system that has no memory in it. For this, we randomised (i.e., induced τ = 0yrs) each of the 5,000 Δ_{47} residual realizations and calculated S(a). Supplementary Figures 9-11d shows the 97.5th, 50th, 2.5th percentiles for the 5,000 instances that S(a) was calculated before (blue) and after (red) residuals were randomised. For all the cores the two systems have similar behaviours implying that τ, and in turn the autocorrelation, in the residuals is very small. This is corroborated when looking at the kernel density estimate of τ (Supplementary Fig. 9-11e), a rough estimate of the memory in the residuals, which is always <1000years.

(c) The heteroscedasticity was evaluated by checking that the squared residuals[11] were not autocorrelated, following the same procedure explained in *(b)*. Supplementary Figs. 9-11f-g show that, for all three sediment cores, the squared residuals show no autocorrelation.

References

1. Zaarur, S., Affek, H. P. & Brandon, M. T. A revised calibration of the clumped isotope thermometer. *Earth and Planetary Science Letters* **382**, 47-57, DOI:10.1016/j.epsl.2013.07.026 (2013).
2. Martrat, B., Jimenez-Amat, P., Zahn, R. & Grimalt, J. O. Similarities and dissimilarities between the last two deglaciations and interglaciations in the North Atlantic region. *Quaternary Science Reviews* **99**, 122-134, DOI:10.1016/j.quascirev.2014.06.016 (2014).
3. Marino, G. *et al.* Bipolar seesaw control on last interglacial sea level. *Nature* **522**, 197-201, DOI:10.1038/nature14499 (2015).
4. Krzywinski, M. & Altman, N. Points of Significance: Visualizing samples with box plots. *Nat Meth* **11**, 119-120, DOI:10.1038/nmeth.2813 (2014).
5. Spitzer, M., Wildenhain, J., Rappsilber, J. & Tyers, M. BoxPlotR: a web tool for generation of box plots. *Nat Meth* **11**, 121-122, DOI:10.1038/nmeth.2811 (2014).
6. Tukey, J. (Addison-Wesley, 1977).
7. Grant, K. M. *et al.* Rapid coupling between ice volume and polar temperature over the past 150,000years. *Nature* **491**, 744-747, DOI:10.1038/nature11593 (2012).
8. Jarque, C. M. & Bera, A. K. Efficient tests for normality, homoscedasticity and serial independence of regression residuals. *Economics Letters* **6**, 255-259, DOI:10.1016/0165-1765(80)90024-5 (1980).
9. Lilliefors, H. W. On the Kolmogorov-Smirnov Test for Normality with Mean and Variance Unknown. *Journal of the American Statistical Association* **62**, 399-402, DOI:10.2307/2283970 (1967).
10. Mudelsee, M. TAUEST: a computer program for estimating persistence in unevenly spaced weather/climate time series. *Computers & Geosciences* **28**, 69-72 (2002).
11. Li, W. K. & Mak, T. K. ON THE SQUARED RESIDUAL AUTOCORRELATIONS IN NON-LINEAR TIME SERIES WITH CONDITIONAL HETEROSKEDASTICITY. *Journal of Time Series Analysis* **15**, 627-636, DOI:10.1111/j.1467-9892.1994.tb00217.x (1994).
12. Grant, K. M. *et al.* The timing of Mediterranean sapropel deposition relative to insolation, sea-level and African monsoon changes. *Quaternary Science Reviews* **140**, 125-141, DOI:10.1016/j.quascirev.2016.03.026 (2016).
13. Grant, K. M. *et al.* A 3 million year index for North African humidity/aridity and the implication of potential pan-African Humid periods. *Quaternary Science Reviews* **171**, 100-118, DOI:10.1016/j.quascirev.2017.07.005 (2017).
14. Rohling, E. J., Marino, G. & Grant, K. M. Mediterranean climate and oceanography, and the periodic development of anoxic events (sapropels). *Earth-Science Reviews* **143**, 62-97, DOI:10.1016/j.earscirev.2015.01.008 (2015).
15. Rohling, E. J. *et al.* Reconstructing past planktic foraminiferal habitats using stable isotope data: a case history for Mediterranean sapropel S5. *Marine Micropaleontology* **50**, 89-123, DOI:10.1016/S0377-8398(03)00068-9 (2004).
16. Berger, A. Long-Term Variations of Daily Insolation and Quaternary Climatic Changes. *Journal of the Atmospheric Sciences* **35**, 2362-2367, DOI:10.1175/1520-0469(1978)035<2362:ltvodi>2.0.co;2 (1978).

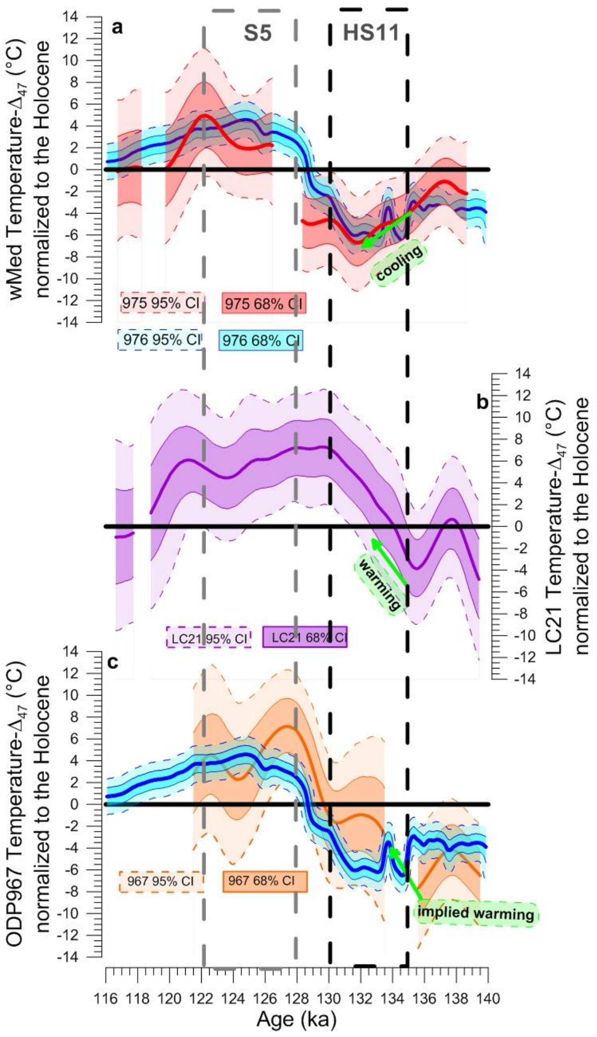

Supplementary Figure 1. TII and last interglacial temperature gradient with respect to late-Holocene in the wMed and eMed (individual records). Gradients were calculated by subtracting the late-Holocene Δ_{47}-temperature average values (~17±2 °C for wMed and ~22±2 °C for eMed records) from each of the 5,000 simulations of **a**, (in red) ODP975; **b**, LC21; and **c**, ODP967Δ_{47}-temperature records, respectively. In **a** and **c** we also show the $U^{K'}_{37}$-temperature record from ODP976 normalized (blue) to the late-Holocene temperature value obtained from ref.[2]. Light and dark shadings correspond to the 95 % and 68 % CI of the temperature gradients. Dashed grey and black boxes highlight the last interglacial S5 interval[7] (~128.3-121.5 ka) and HS11 (135-130 ka, ref. 3), respectively. Gaps in the final Δ_{47}-records correspond to intervals where the age uncertainties of the Δ_{47}-replicates do not overlap.

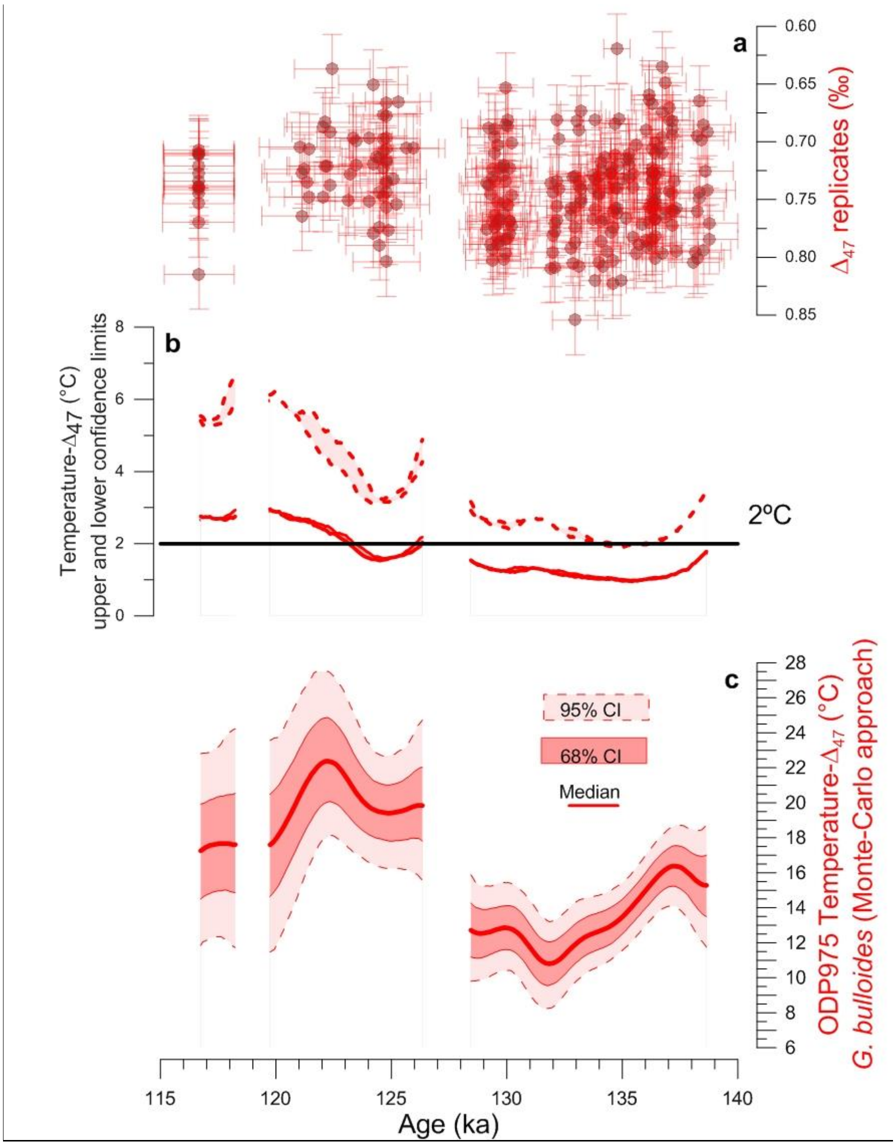

Supplementary Figure 2. Temperature error across the ODP975 *G. bulloides* record. a, Δ_{47}-replicates. **b,** Upper and lower limits of the 68 % CI (thick lines) and 95 % CI (dashed lines) subtracted from the median in the Δ_{47}-temperature ODP975 record shown in **c**. Grey boxes highlight the gaps in the Δ_{47}-replicates data and the effect in the errors. Notice that our data-processing approach accounts for intervals of low Δ_{47}-replicates density that result from low-foraminifera abundances. The confidence intervals become larger (smaller) during periods of low (high) Δ_{47}-replicates density. Gaps in the final Δ_{47}-record correspond to intervals where the age uncertainties of the Δ_{47}-replicates do not overlap.

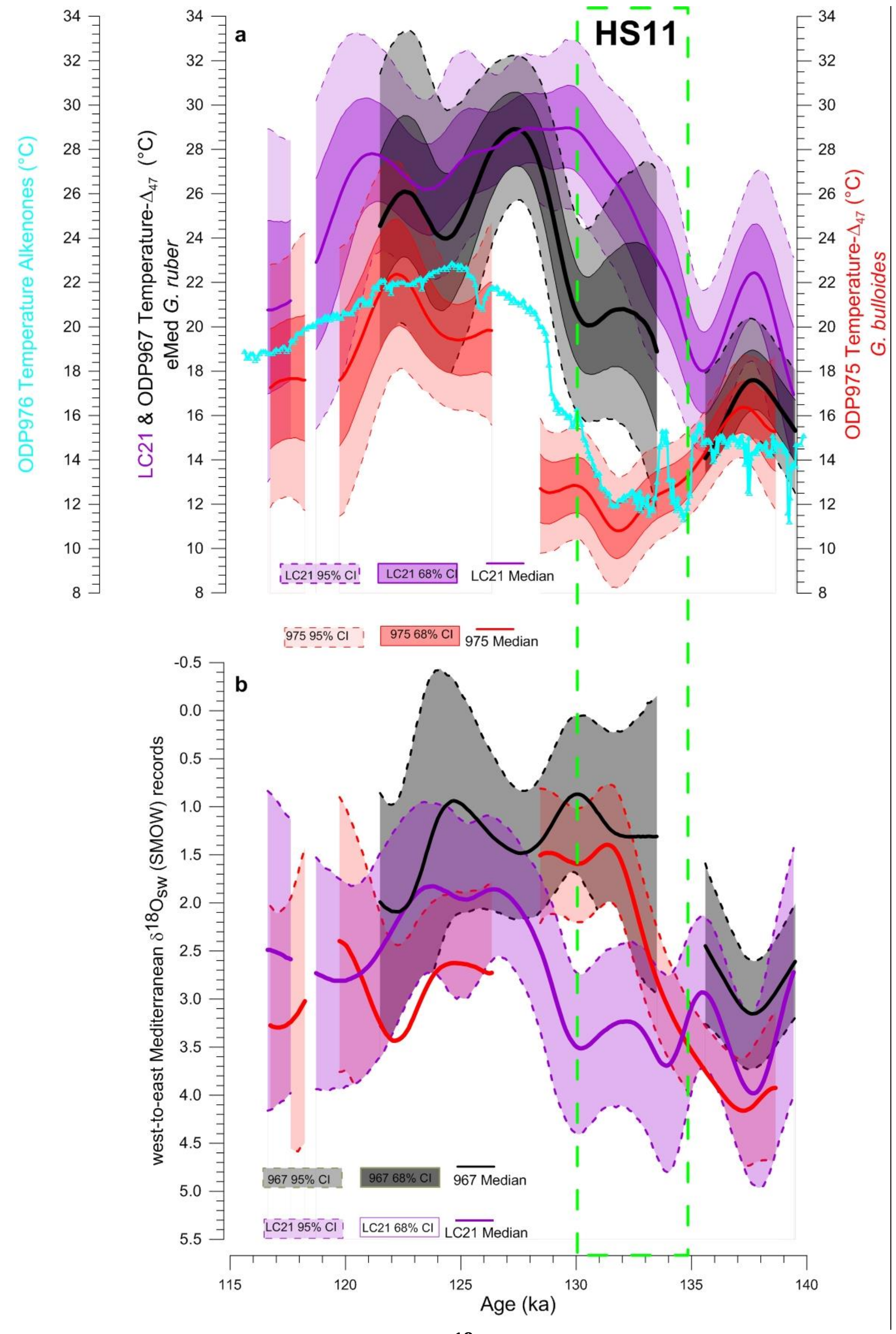

Supplementary Figure 3. Temperature and $\delta^{18}O_{SW}$ contrast across the Mediterranean Sea during HS11. Thick lines correspond to the median of 5,000 Monte Carlo simulations of the Δ_{47}-replicates and ages within their (1σ) uncertainties. Light and dark shadings correspond to the 95 % and 68 % CI of the records. Light green box highlights the HS11 (135-130 ka, ref. [3]). Gaps in the final Δ_{47}-records correspond to intervals where the age uncertainties of the Δ_{47}-replicates do not overlap. In panel b we only shoe the 95 % CI of the $\delta^{18}O_{SW}$ records.

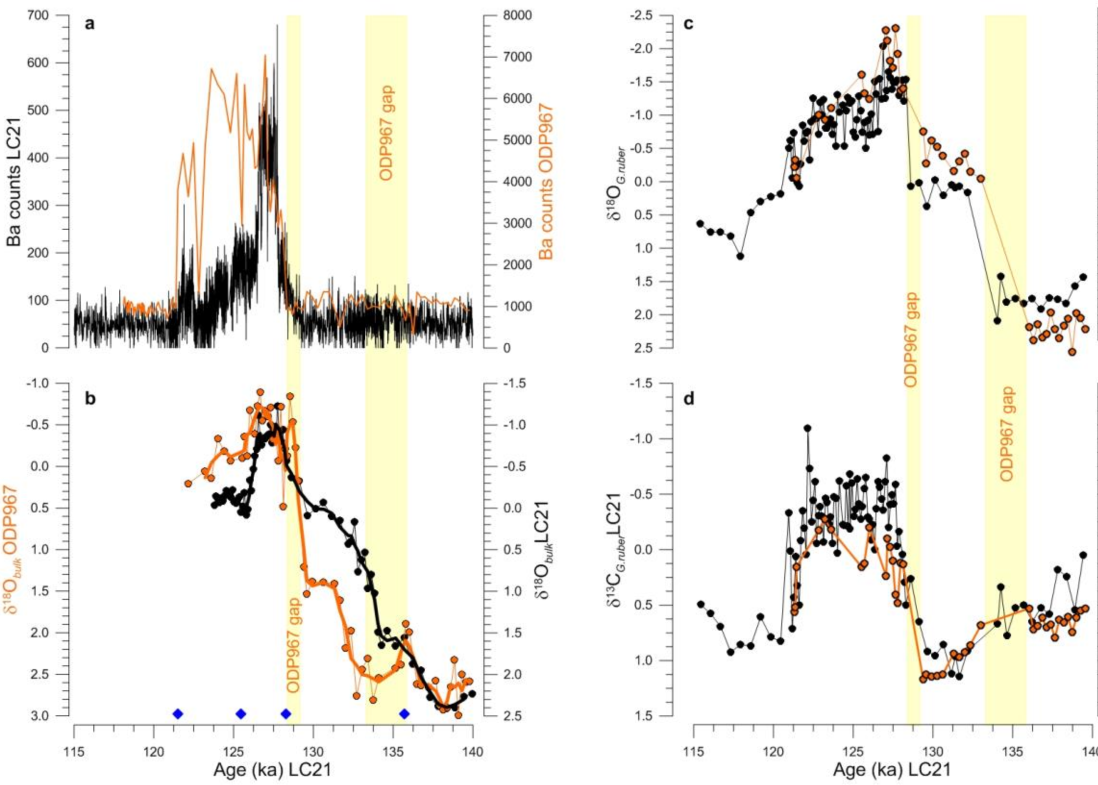

Supplementary Figure 4. ODP967 age model. a, XRF-Ba counts from LC21 (ref. 12) (black) and ODP967 (ref.[13,14]) (orange). **b,** Bulk δ^{18}O records from LC21 (black) and ODP967 (orange). Blue diamonds in **b** are the tie points used to tune the ODP967 to LC21 record from (ref. [7]). Comparison of a high temporal resolution *G. ruber* $\delta^{18}O_C$ **c,** and $\delta^{13}C_C$ **d,** record from LC21 (ref. [7]) and ODP967 on the LC21 chronology. Yellow boxes highlight the gaps in ODP967 *G. ruber* δ^{18}O record that complicated the synchronisation across TII.

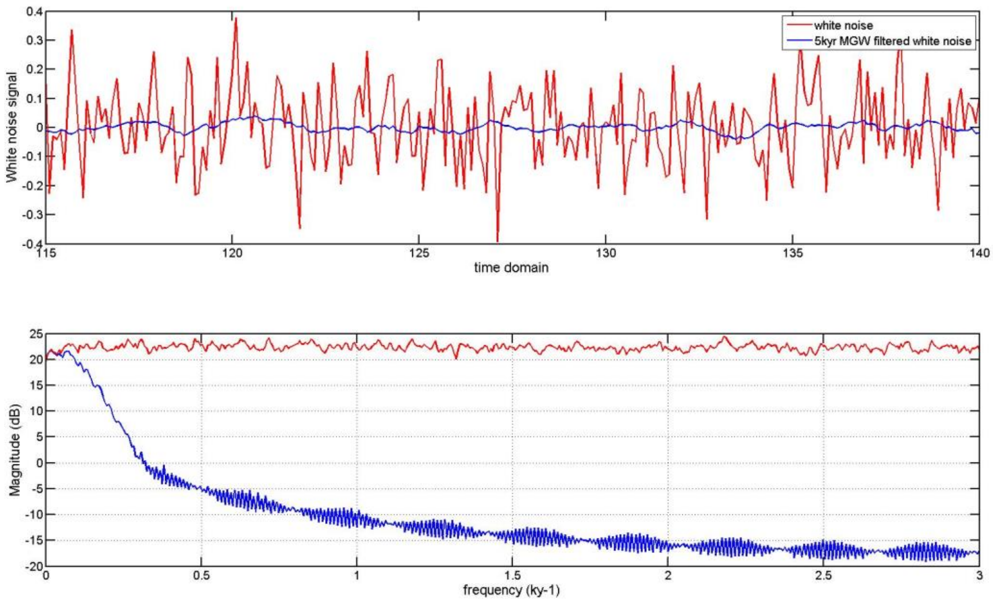

Supplementary Figure 5. Frequency response of a 5 kyr Moving Gaussian Window (MGW) filter. a, random (white) noise time series before (red) and after (blue) applying the 5 kyr MGW filter. **b**, frequencies in the random (white) noise time series before (red) and after (blue) applying the 5 kyr MGW filter. Observe how the high frequencies are attenuated after applying the filter.

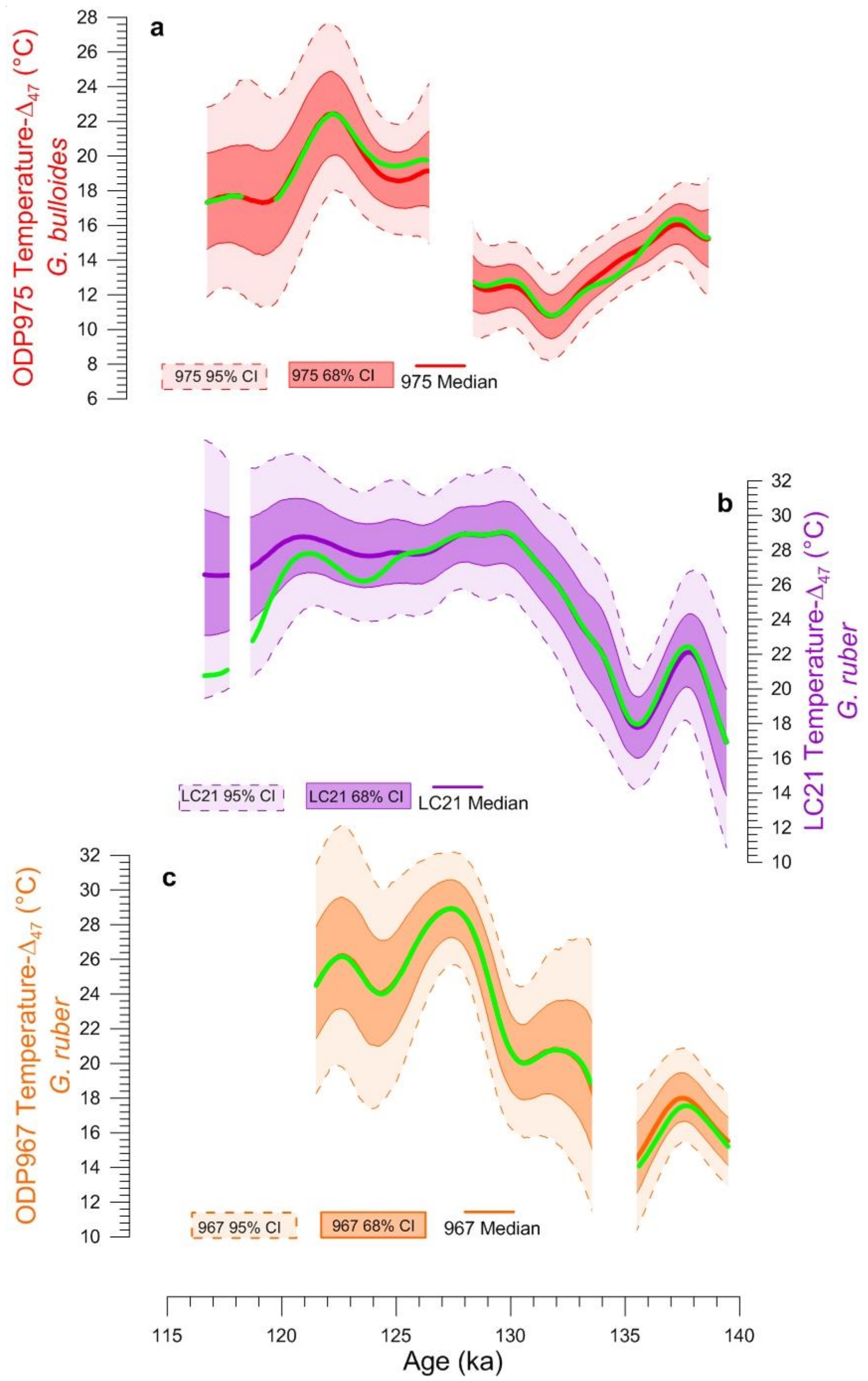

Supplementary Figure 6. Δ_{47}-temperature records when outlier test uses W = 1.5. a, ODP975. **b,** LC21 and **c,** ODP967. Light and dark shadings correspond to the 95 % and 68 % CI of the records. For comparison, we show in green the median of each record using the conservative outlier test (same records shown in the main figures). Gaps in the final Δ_{47}-records correspond to intervals where the age uncertainties of the Δ_{47}-replicates do not overlap.

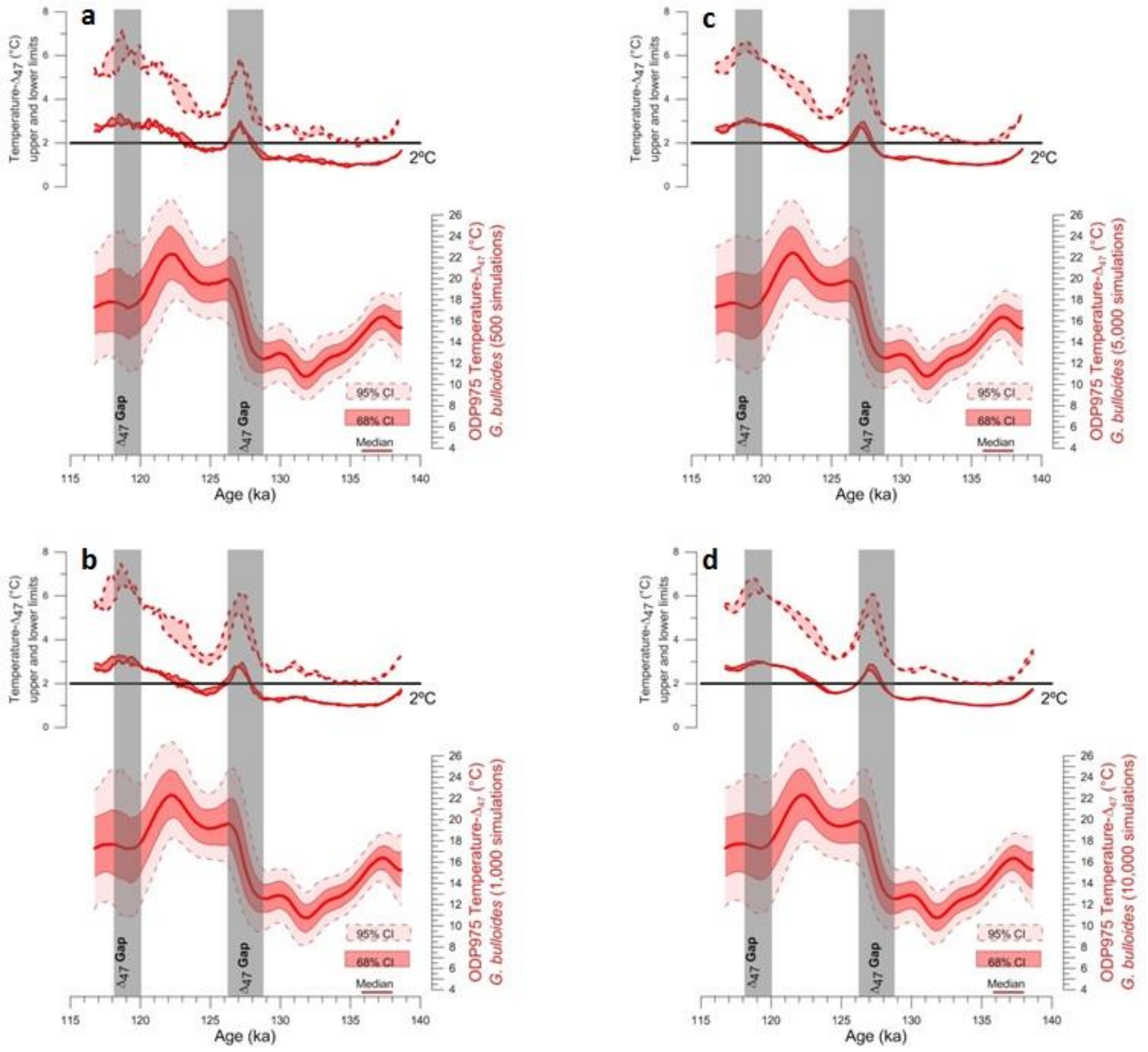

Supplementary Figure 7. Effect of the number of Monte Carlo simulations on the final ODP975 Δ_{47}-temperature record. a, 500**; b,** 1,000; **c,** 5,000; **d,**10,000 simulations . Light and dark shadings correspond to the 95 % and 68 % CI of the records. Note that the shape of the record is not affected, but the confidence intervals are less smooth as the number of simulations decrease. In grey we highlight the gaps in the Δ_{47}-records. Observe how the data processing approach accounts for these gaps by increasing the confidence intervals. However, for avoiding over-interpretation of the data we have removed from the main figures the intervals where the age uncertainties of the Δ_{47}-replicates do not overlap.

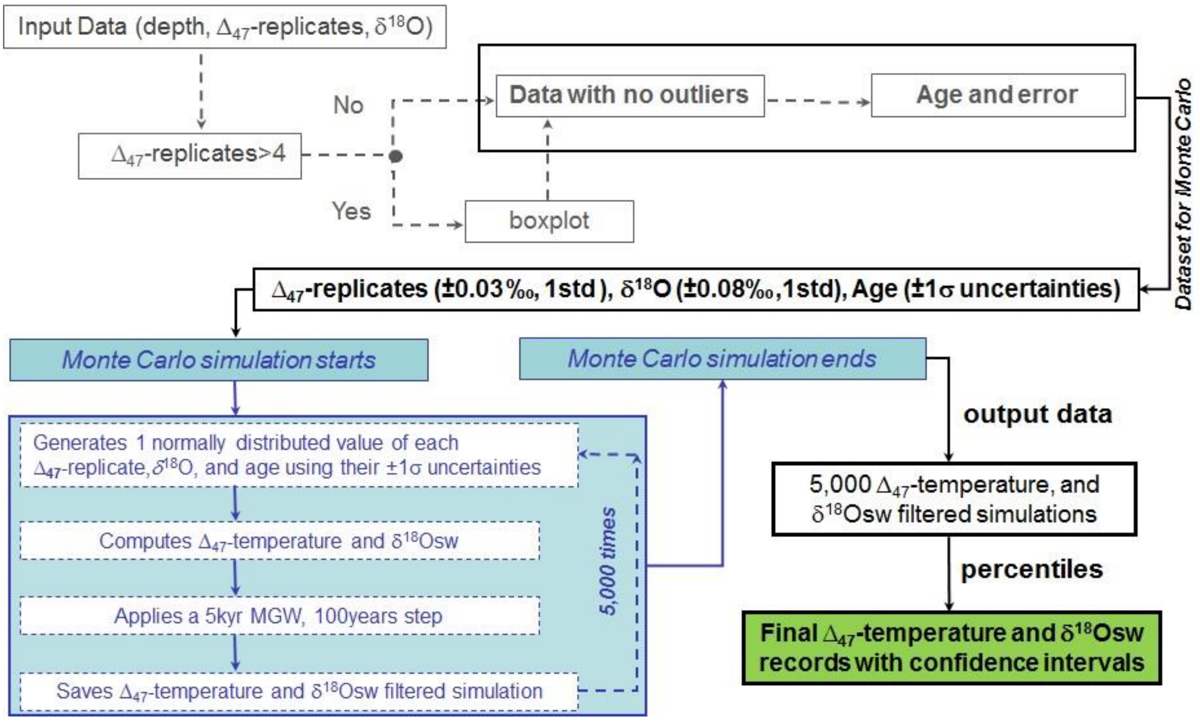

Supplementary Figure 8. Flow chart summarizing the steps of the non-traditional data-analysis approach.

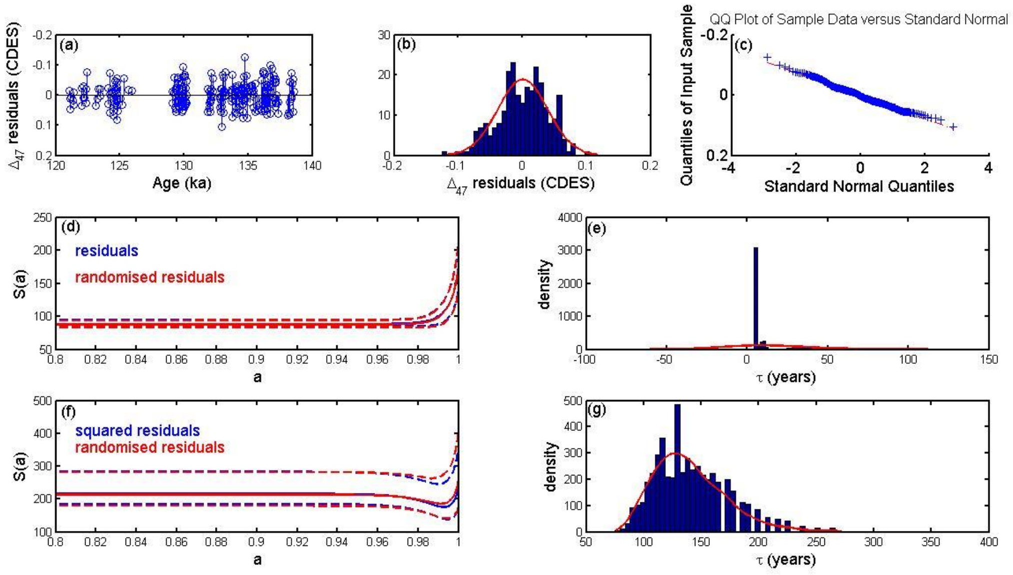

Supplementary Figure 9. Residuals analysis for the Δ_{47}-record from ODP975. In the upper panels **(a-c)** we check that residuals are normally distributed, in the middle panels **(d-e)** we check that residuals are not auto-correlated (persistence time τ<1,000year), and in the lower panels **(f-g)** we check residuals for conditional heteroscedasticity. **a,** stem plot of the residuals between the measured Δ_{47}-replicates and the 50th percentile of the Δ_{47} MGW filtered Monte Carlo realizations. **b**, histogram of the residuals. **c**, Quantile-quantile plot of the residuals. **d**, variation of S(a) - the least-squares estimator that minimizes *a* (*a* = exp(-1/τ))- when *a* varies from 0.8 to 1 and we use the residuals (blue) or the randomised residuals (red). **e**, kernel density estimate of τ. **f-g** are like **d-e**, respectively, but using the squared-residuals.

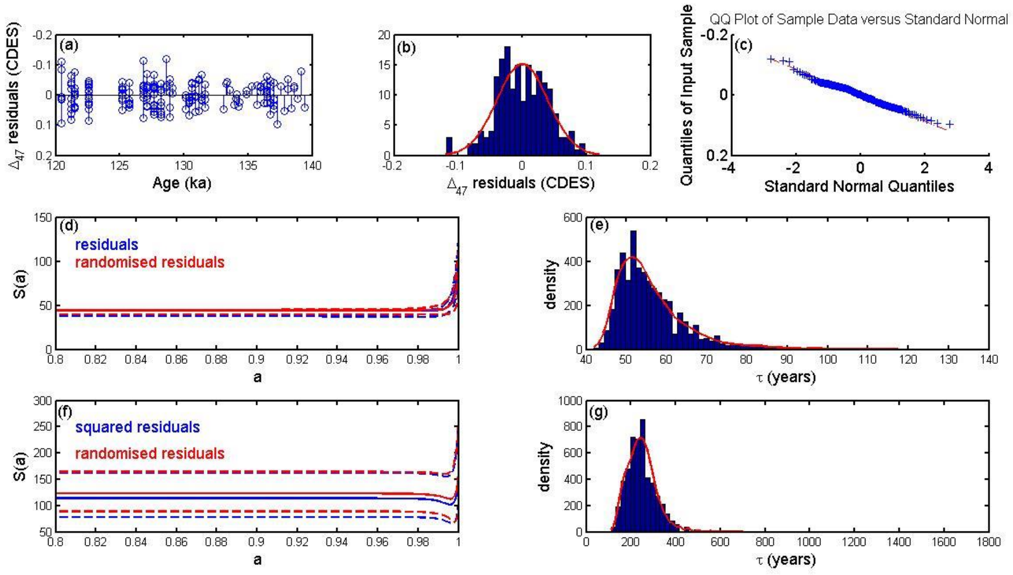

Supplementary Figure 10. Residuals analysis for the Δ_{47}- record from LC21. In the upper panels **(a-c)** we check that residuals are normally distributed, in the middle panels **(d-e)** we check that residuals are not auto-correlated (persistence time τ<1,000year), and in the lower panels **(f-g)** we check residuals for conditional heteroscedasticity. **a,** stem plot of the residuals between the measured Δ_{47}-replicates and the 50th percentile of the Δ_{47} MGW filtered Monte Carlo realizations. **b**, histogram of the residuals. **c**, Quantile-quantile plot of the residuals. **d**, variation of S(a) - the least-squares estimator that minimizes *a* (a = exp(-1/τ))- when *a* varies from 0.8 to 1 and we use the residuals (blue) or the randomised residuals (red). **e**, kernel density estimate of τ. **f-g** are like **d-e**, respectively, but using the squared-residuals.

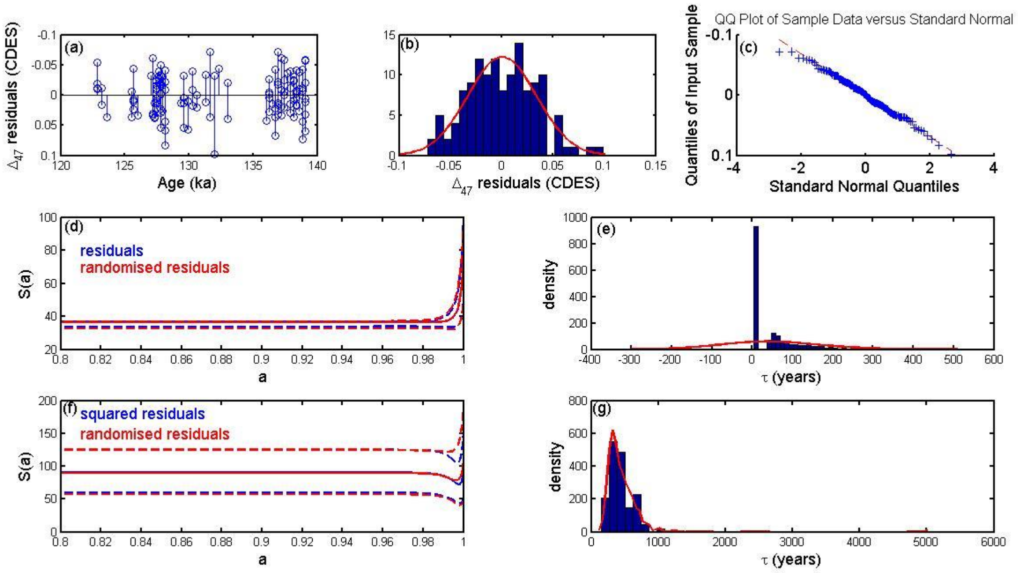

Supplementary Figure 11. Residuals analysis for the Δ_{47}- record from ODP967. In the upper panels **(a-c)** we check that residuals are normally distributed, in the middle panels **(d-e)** we check that residuals are not auto-correlated (persistence time τ<1,000year), and in the lower panels **(f-g)** we check residuals for conditional heteroscedasticity. **a,** stem plot of the residuals between the measured Δ_{47}-replicates and the 50th percentile of the Δ_{47} MGW filtered Monte Carlo realizations. **b**, histogram of the residuals. **c**, Quantile-quantile plot of the residuals. **d**, variation of S(a) - the least-squares estimator that minimizes *a* (*a* = exp(-1/τ))- when *a* varies from 0.8 to 1 and we use the residuals (blue) or the randomised residuals (red). **e**, kernel density estimate of τ. **f-g** are like **d**-**e**, respectively, but using the squared-residuals.

Supplementary Table 1. Insolation-induced extra warming in the summer mixed layer during the onset of S5 with respect to late-Holocene. Summer mixed layer value at present is ~50m and it is expected to shoal during sapropel periods[15]. Insolation was obtained from ref. [16] using AnalySeries software. We averaged summer (June 21st) and winter insolation (December 21st) at 34°N during the late-Holocene (0-2 ka) and onset of the S5 (127-129 ka). We calculated the resultant mixed layer heating for three scenarios: a) using late-Holocene insolation and present mixed layer; b) onset of S5 insolation and present mixed layer; c) onset of S5 insolation and shallower mixed layer (35 m). Attenuation (a) in all cases was varied between 0.4-0.6 (20 % of that in Athens).

				Surface water heating (°C)			S5-Holocene gradient (°C)		
	Insolation (W/m^2)	variability (W/m^2)	mixed layer (m)	a=0.4	a=0.5	a=0.6	a=0.4	a=0.5	a =0.6
**a) Holocene**	340	150	50	15.8	12.7	9.5	-	-	-
**b) S5 onset**	358	180	50	17.5	14.0	10.5	1.7	1.3	1.0
**c) S5 onset**	358	180	35	25.0	20.0	15.0	9.2	7.3	5.5

Supplementary Table 2. Tie points for the ODP967 age model based on the radiometrically constrained chronology[7] for LC21

ODP967 depth (m)	Age (ka) synchronized to LC21	±1σ error age	tie point from
5.50	107.80	0.54	XRF
7.50	121.50	1.71	XRF
7.61	125.48	1.36	δ^{18}O bulk
7.80	128.30	1.08	XRF
8.02	135.69	0.79	δ^{18}O bulk
9.72	175.63	1.00	XRF

Supplementary Table 3.Carbonate standards values for data processing

	δ^{13}C (‰, VPDB)	δ^{18}O (‰, VPDB)	Δ_{47} (‰, CDES)
**ETH-1**	2.0	-2.17	0.265
**ETH-2**	-10.2	-18.59	0.267
**ETH-3**	1.67	-1.76	0.703
**ETH-4**	-10.22	-18.66	0.522
